# Elements of Trust in Digital Health Systems: Scoping Review

**DOI:** 10.2196/11254

**Published:** 2018-12-13

**Authors:** Afua Adjekum, Alessandro Blasimme, Effy Vayena

**Affiliations:** 1 Department of Health Sciences and Technology ETH Zurich Zurich Switzerland

**Keywords:** digital health, digital health technologies, health care, health systems, trust

## Abstract

**Background:**

Information and communication technologies have long become prominent components of health systems. Rapid advances in digital technologies and data science over the last few years are predicted to have a vast impact on health care services, configuring a paradigm shift into what is now commonly referred to as digital health. Forecasted to curb rising health costs as well as to improve health system efficiency and safety, digital health success heavily relies on trust from professional end users, administrators, and patients. Yet, what counts as the building blocks of trust in digital health systems has so far remained underexplored.

**Objective:**

The objective of this study was to analyze what relevant stakeholders consider as enablers and impediments of trust in digital health.

**Methods:**

We performed a scoping review to map out trust in digital health. To identify relevant digital health studies, we searched 5 electronic databases. Using keywords and Medical Subject Headings, we targeted all relevant studies and set no boundaries for publication year to allow a broad range of studies to be identified. The studies were screened by 2 reviewers after which a predefined data extraction strategy was employed and relevant themes documented.

**Results:**

Overall, 278 qualitative, quantitative, mixed-methods, and intervention studies in English, published between 1998 and 2017 and conducted in 40 countries were included in this review. Patients and health care professionals were the two most prominent stakeholders of trust in digital health; a third—health administrators—was substantially less prominent. Our analysis identified cross-cutting personal, institutional, and technological elements of trust that broadly cluster into 16 enablers (altruism, fair data access, ease of use, self-efficacy, sociodemographic factors, recommendation by other users, usefulness, customizable design features, interoperability, privacy, initial face-to-face contact, guidelines for standardized use, stakeholder engagement, improved communication, decreased workloads, and service provider reputation) and 10 impediments (excessive costs, limited accessibility, sociodemographic factors, fear of data exploitation, insufficient training, defective technology, poor information quality, inadequate publicity, time-consuming, and service provider reputation) to trust in digital health.

**Conclusions:**

Trust in digital health technologies and services depends on the interplay of a complex set of enablers and impediments. This study is a contribution to ongoing efforts to understand what determines trust in digital health according to different stakeholders. Therefore, it offers valuable points of reference for the implementation of innovative digital health services. Building on insights from this study, actionable metrics can be developed to assess the trustworthiness of digital technologies in health care.

## Introduction

### Background

Digital health broadly refers to the use of information and communication technologies to improve human health, health care services, and wellness for both individuals and populations [1,2]. It has been argued that the capacity to collect, store, and analyze extensive amounts of health data is the chief driving force of digital health [3]. The accessibility of such data is rejuvenating the process involved in diagnosing, managing, and treating disease, thus exceeding the conventional boundaries of how health care institutions and providers operate. A case in point is the myriad number of smartphone apps that allow patients to seamlessly monitor various aspects of their health care beyond the confines of a health care institution [1].

There is currently no consensus on a definition for digital health. The term “digital medicine” for instance, resembles digital health, as it also refers to the use of digital technologies such as biosensors and smartphones to refine and individualize medicine [4]. Given how they are often described, electronic health, mobile health (mHealth), telecare, and telehealth could also be used interchangeably with digital health [5]. This ambiguity calls for a need to generate an inclusive definition that captures the different terms that may be used to portray digital health.

The US Food and Drug Administration (FDA) depicts digital health as comprising of mHealth, wearable devices, telehealth, telemedicine, personalized medicine, electronic health records (EHRs), and health information technology (IT) [6]. In this review, we adopt this as our working definition of digital health. Throughout this paper, the term “digital health” refers to all of the aforementioned categories. So far, there has been a prolific development of digital health technologies, and the value of such ventures continues to rise at a steady pace. In 2017 alone, the global net worth of the digital health industry was estimated at US $25 billion (£19 billion; €21 billion). Some estimates even project that digital health could cut back up to US $7 billion of US health care expenditure annually [7].

Beyond economic gains, improved safety and efficacy are among the anticipated benefits of digital health [7-10]. Current evidence supports the notion that digital health does indeed bolster safety within health systems [11]. In the domain of health care delivery, digital health promises to abate mortality, shorten hospital admissions, and decrease medication errors [11]. Despite these advances, there are privacy and data protection concerns associated with the pace of development of digital health products [7,12]. Moreover, as data from digital health tools such as mHealth apps increasingly inform medical decision making, the issue of medical liability comes to the fore [13,14]. The considerations about privacy and data protection highlight the ethical challenges that bear directly on the trustworthiness of digital health. While numerous studies have analyzed such ethical issues [15-19], the determinants of trust in digital health are yet to receive comparable levels of attention [1,3,20-22].

### What is Trust?

Trust is an elusive concept that is difficult to pin down in operational terms. Relationships of trust can exist between individuals, between individuals and the organizations they come into contact with, or between 2 organizations of any given nature [23]. Trust is oftentimes illustrated as a relationship between one party (a trustor) and another (a trustee) with optimistic anticipation that the trustee will fulfill the trustor’s expectations [23,24]. Trust relationships often lack enforceable obligations and are thus vulnerable to deception [25]. Consequently, different sets of reasons encourage trust relationships. Chief among them are the trustee’s reliability (possessing a good reputation), competence (having the technical skills to perform the task at hand), and integrity (generally acting in an honest way) [26].

Within health systems, trust is a prominent component of doctor-patient relationships [27-29]. It improves not only health care access but also treatment outcomes and patient satisfaction [30,31]. However, whether or not it is appropriate to talk about trust between people and inanimate objects—such as technological products—remains an open question in the literature [21,32]. Indeed, the inclination of individuals to purchase or use products that are derived from “expert systems”—those structures that rely on either technical know—how or professional expertise and whose outcomes are consequently pervasive, opaque, or easily taken for granted—has been described as a tangible component of trust [33].

Some experts suggest that trust is propelled by contingency rather than risk, while others maintain that the ability to weigh risks and to choose between different actions drives trust [34]. Despite the risk of deception within any trust relationship, it is disputable whether one chooses to trust solely by weighing risks or actively by evaluating alternative options. Be that as it may, in the case of medical technologies, institutional trust and technical reliability are deeply intertwined [35]. In terms of digital health technologies, we hypothesize that trust is likely to develop if the risks and uncertainties associated with their use can be minimized.

As health care becomes increasingly dependent on digital technologies, exploring what determines and what foregoes trust in digital health is of paramount importance. Identifying the factors pertinent to trust can inform the development of novel health care services as well as meet the needs and expectations of users and patients. In addition, such factors can be taken into account for the assessment of both new and existing digital health services. Thus, this study seeks to contribute to this discourse by analyzing what the relevant stakeholders in digital health consider as the enablers and impediments of trust in digital health.

## Methods

### Overview

This review aimed to summarize the enabling and impeding factors of trust in digital health. To this end, we conducted a scoping review using Arksey and O’Malley’s proposed framework on scoping reviews [36]. A scoping review methodology was chosen, as it appropriately captures broad and ambiguous topics, like digital health, that may involve a myriad of study designs. We searched for studies that reported on the perspectives of different digital health stakeholders. From these perspectives, we discerned views on what was reported to facilitate trust and what hindered it. Often, some of these same factors were recognized as relevant for the acceptance of a particular technology. By acceptance, we mean adoption and use grounded in or at least co-occurring with trust on the part of users. This understanding of trust as a potential determinant of acceptance reflects some credited models of technology acceptance in the health care sector [37].

### Information Sources

We searched 5 databases: MEDLINE, EMBASE, the Cumulative Index to Nursing and Allied Health Literature, PsycINFO, and Web of Science for peer-reviewed studies as well as gray literature. We worked with a research librarian at the University of Zurich, Switzerland, to identify relevant bibliographic databases and to construct a search strategy that would ensure comprehensive results.

### Search Strategy

The search strategy involved formulating keywords and Medical Subject Headings around the 2 main themes of this study, namely, trust and digital health. Since the concept of trust can be ill-defined within the literature [35], we set out to include synonyms such as expectation, mistrust, confidence, and experience to capture the heterogeneity of trust descriptions within the literature (Multimedia Appendix 1). Digital health, on the other hand, was disaggregated into its distinctive components as described by the FDA: mHealth, wearable devices, telehealth, telemedicine, personalized medicine, and health IT. The searches were restricted to publications available in English, French, German, Italian, and Spanish with no publication date restrictions, to allow the search results to encompass a broad range of relevant studies. The searches commenced on July 20, 2017, and concluded on August 18, 2017. The recovered studies were then exported into the Endnote X8.2 reference software.

### Eligibility Criteria of Included Studies

To capture the wide array of studies that may be relevant to this topic, we did not predefine the study designs of included studies. This allowed for the inclusion of qualitative, quantitative, intervention, and mixed-methods studies. We assessed the relevance of the retrieved studies to ensure that they related to either of the abovementioned digital health technologies. Moreover, each study was required to meet at least 1 of the following criteria: (1) investigate stakeholder perceptions, attitudes, expectations, and perspectives toward digital health or (2) highlight some potential enablers and impediments to trust in digital health technologies and services.

### Study Selection, Categorization, and Data Extraction

As is customary in scoping reviews, we employed an iterative approach to select, categorize, and extract data from the recovered studies [36]. We used a 2-step process to select relevant articles. At first, 1 author (AA) reviewed all of the titles and abstracts derived from the search. In order to reduce sampling bias [38], a second author (AB) reviewed a random sample of 243 titles along with their associated abstracts (constituting 10% of the total sample after duplicates had been removed). To assess the level of agreement between the 2 reviewers, an interrater reliability score using Cohen kappa was computed along with its corresponding CI and *P* value. The Cohen kappa score for the 2 coders (AA and AB) was .661 (95% CI 0.465-0.857; *P*<.001). According to McHugh (2012), a kappa of.661 signifies a moderate agreement between the coders [39].

**Figure 1:**
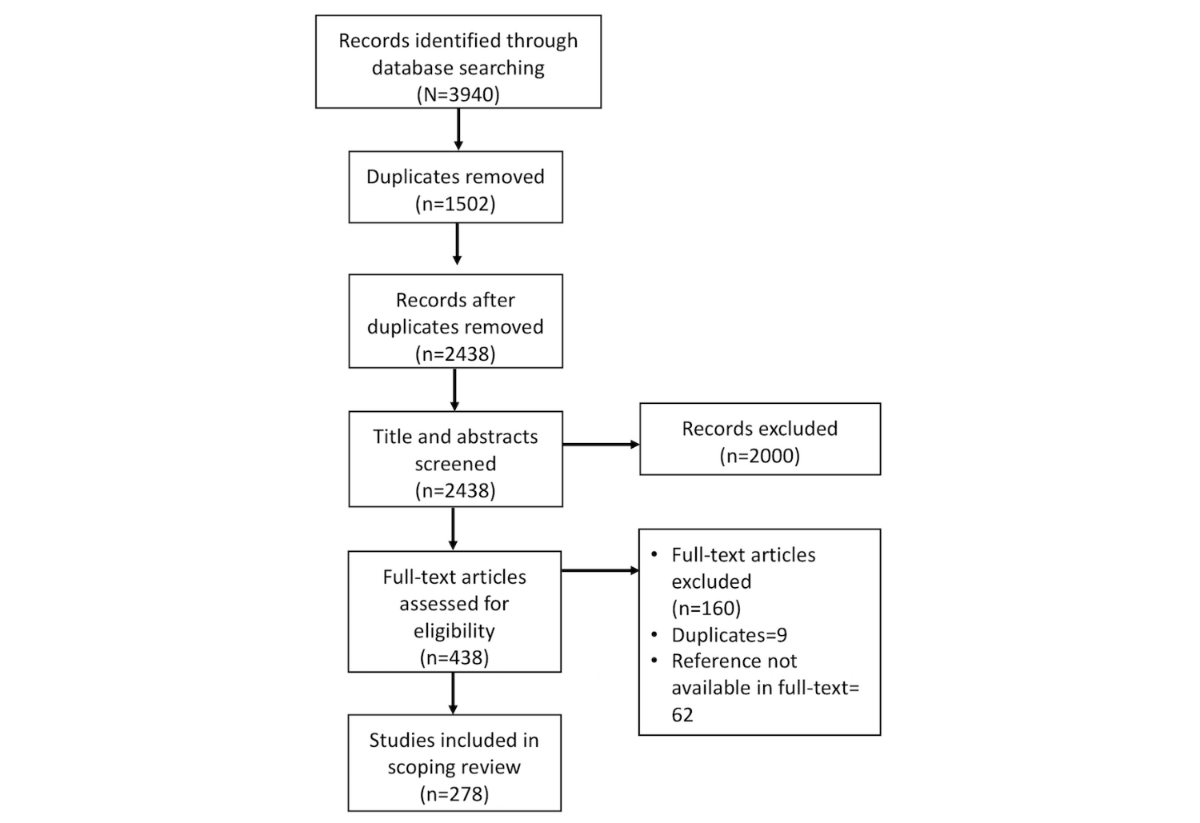
Preferred Reporting Items for Systematic Reviews and Meta-Analyses (PRISMA) flow diagram.

Overall, we retrieved a total of 3940 search results from the 5 databases. Of these, 1474 were identified as duplicates and discarded. However, during the screening process, we discovered an extra 28 duplicates, increasing the total number discarded to 1502. This led to screening the titles and abstracts of 2438 articles of which 438 were eligible for full-text screening. The Preferred Reporting Items for Systematic Reviews and Meta-Analyses flow diagram below (Figure 1) lays out these procedures in more detail [40]. The final number of articles included in the review was 278.

From each article, we documented the author’s name, year of publication, country of origin, sample size, study design (eg, qualitative or quantitative), digital health type as well as the relevant stakeholders. A descriptive, analytical approach was used to summarize the outcomes of the studies. We identified the trust elements (enablers and impediments) by charting the key themes and issues identified from each study [36]. To develop these themes, the results section of each study was scrutinized to identify various stakeholder priorities, perspectives, expectations, perceptions, and attitudes toward a particular digital health technology or service. Multimedia Appendix 2 shows the studies from which each element was derived. Since either an enabler or impediment could be derived from the same study, we reported the overall number of studies that support each element rather than percentages. Simultaneously, we compiled a list of recurring terminologies that were used to represent or describe the various digital health technologies, which we termed “health technology types.”

## Results

### Characteristics of Articles

Of the 278 articles included in this review, 51 (51/278, 18.3%) related to telemedicine and telehealth, 24 (24/278, 8.6%) to personalized medicine, 47 (47/278, 16.9%) to mHealth, 73 (73/278, 26.3%) to health IT, 73 (73/278, 26.3%) to EHRs, and 4 (4/278, 1.4%) to wearable devices, while 6 (6/278, 2.2%) concerned 2 or more digital health technologies. Most of the studies were conducted in 2015 (50/278, 18.0%), and the median year was 2014. The oldest study was conducted in 1998, while the most recent study was from 2017. There were 98 qualitative studies, 133 quantitative studies, 45 mixed method studies, and 2 intervention studies. Data from Web-based sources were collected in 7 studies. Overall, the studies were conducted in 40 countries; the United States was the most represented (101/278, 36.3%). The United Kingdom had the second highest number of studies (47/278, 16.9%) followed by Australia (16/278, 5.8%) and Canada (15/278, 5.4%; see Multimedia Appendix 3).

### Digital Health Technologies and Services

For each digital health technology, we uncovered several health technology types employed to provide digital health services. Within each digital health category, there appear to be multiple terminologies to describe identical or variable technologies or services. In many instances, there were only slight variations differentiating one service from the other. For example, electronic patient records, electronic medical records, and electronic health care records were variable forms of EHRs, while Web-based consultations, online support groups, and Web-based health information were some examples of health IT. Multimedia Appendix 4 provides a list of the variable terminologies identified from the included studies.

### Stakeholders

In our analysis, we identified 2 major stakeholders: *patients* or the *public* (187 studies) and *health care professionals* (HCPs; 101 studies). A third less predominant group—*health administrators* (HAs; 20 studies)—was also identified. For the sake of clarity, HCPs refer to a broad range of health care specializations that include pharmacists, occupational therapists, physical therapists, physicians, and nurses. Other stakeholders that were considerably less represented in the analyzed studies included medical and nursing students, consumer groups, health policy makers, data controllers, academic researchers, social workers, counselors, and IT technicians.

### Trust Enablers and Impediments

Our findings indicate that trust in digital health technologies and services is affected by a variety of elements. In this study, trust enablers refer to those factors that encourage stakeholders’ trust in digital health, while trust impediments denote the factors that can potentially hinder trust. These trust enablers and impediments, therefore, underscore the elements that influence stakeholder decisions on whether or not to place their trust in digital health technologies.

#### Personal Elements

By personal elements, we designate factors that influence trust in digital health at the individual level. The higher the likelihood of a digital health technology or service to enhance job performance, the more likely stakeholders are to trust it due to convenience and *usefulness* (110 studies). Moreover, *sociodemographic factors* (84 studies) such as ethnicity, income, and educational status affected an individual’s trust in digital health either positively or negatively, thereby acting simultaneously as enablers and impediments. *Ease of use* (53 studies)—the propensity for systems to require minimal effort for use—also influenced trust positively. Other personal elements include *fair data access* (21 studies), *recommendations* (17 studies) from family members, acquaintances and colleagues as well as *self-efficacy* (15 studies). The latter denotes a refined acumen to manage one’s own health [41]. *Altruism* (9 studies) also contributed to stakeholder involvement in digital health enterprises and was driven by the prospect of contributing to novel and beneficial therapies that would benefit society.

A number of studies reported *excessive costs* (34 studies) and *limited accessibility* (55 studies) as potential barriers to trust and, therefore, acceptance. *Fear of data exploitation* (25 studies) from third parties such as insurance and pharmaceutical companies was another palpable impediment to trusting digital health systems.

#### Technological Elements

The technological elements refer to the technical components of digital health technologies that make them appealing to accept and use. In terms of sensitive personal data such as genetic data, robust systems that delivered on safety and *privacy* (73 studies) were crucial to trust. There was a high affinity for *customizable design features* (28 studies) that allowed stakeholders to tailor devices to their specific needs. Since HCPs were often required to utilize disparate software programs, they requested *interoperable* (10 studies) systems that ensured that newer systems are compatible with currently existing ones. Relating to trust impediments, *defective technology* (32 studies) was a culprit for the minimal use of digital health technologies or services.

#### Institutional Elements

The institutional elements denote the strategies that are implemented within establishments that influence stakeholder trust in digital health. Several studies highlighted that various stakeholders had suggestions, expectations, or feedback to provide on how best to improve digital health services. Consequently, *stakeholder engagement* (71 studies), which involves taking stakeholders’ opinions into account, emerged as a relevant condition to increase trust in digital health. *Improved communication* (46 studies) was a cross-cutting expectation from digital health technologies. Both patients and HCPs valued the many communication avenues that digital health provided. In 40 studies, it appeared that there was a need for *initial face-to-face* interactions prior to the introduction of digital health services. Generally, stakeholders expected digital health technologies to build upon and improve on existing systems. Hence, they preferred technologies that *decreased workloads* (82 studies).

The *reputation of service providers* (71 studies), however, served as either an enabler or impediment to trust in digital health. A good reputation encouraged trust and vice versa. *Time-consuming* (42 studies) technologies as well as those that provided *information of poor quality* (51 studies) impeded trust. Other impediments identified included *insufficient training* (54 studies) and uncertainties originating from *inadequate publicity* (44 studies) about the capabilities, existence, and risks involved in using digital health. Finally, trust was also hindered by the absence of *guidelines for standardized use* (22 studies).

In Table 1, we provide a summary of these findings and highlight the stakeholders for whom these elements appeared pertinent. In the table, found in parenthesis next to each element are the total number of studies (n). A checkmark is also used to illustrate the respective trust elements that each stakeholder is associated with.

**Table 1:** Trust enablers and impediments alongside their corresponding stakeholders.

Element classification	Enablers of trust	Impediments to trust	Stakeholders		
			Patients	HCPs^a^	HAs^b^
Personal elements	Altruism (n=9)	N/A^c^	✓^d^	N/A	N/A
	Ease of use (n=30)	N/A	✓	✓	✓
	N/A	Excessive costs (n=34)	✓	✓	✓
	Fair data access (n=21)	N/A	✓	✓	N/A
	N/A	Fear of data exploitation (n=25)	✓	N/A	N/A
	Recommendation by others (n=17)	N/A	✓	✓	N/A
	Self-efficacy (n=15)	N/A	✓	✓	N/A
	N/A	Limited accessibility (n=55)	✓	✓	N/A
	Sociodemographic factors (n=84)^e^	Sociodemographic factors (n=84)^e^	✓	✓	N/A
	Usefulness (n=110)	N/A	✓	✓	N/A
Technological elements	Customizable design features (n=28)	N/A	✓	✓	N/A
	N/A	Defective technology (n=32)	✓	✓	✓
	Interoperability (n=10)	N/A	N/A	✓	N/A
	Privacy (n=73)	N/A	✓	✓	N/A
Institutional elements	Decreased workloads (n=83)	N/A	N/A	✓	✓
	Guidelines for standardized use (n 22)	N/A	N/A	✓	✓
	Improved communication (n=46)	N/A	✓	✓	✓
	N/A	Inadequate publicity (n=44)	✓	✓	✓
	Initial face-to-face contact (n=40)	N/A	✓	✓	N/A
	N/A	Insufficient training (n=54)	✓	✓	✓
	N/A	Poor information quality (n=51)	✓	✓	✓
	Service provider reputation (n=71)^e^	Service provider reputation (n=71)^e^	✓	✓	N/A
	Stakeholder engagement (n=71)	N/A	✓	✓	N/A
	N/A	Time-consuming (n=42)	N/A	✓	✓

^a^HCP: health care professional.

^b^HA: health administrator.

^c^N/A: not applicable.

^d^Check mark indicates respective trust elements that each stakeholder is associated with.

^e^These elements (sociodemographic factors and service provider reputation) are simultaneously trust enablers and impediments.

## Discussion

### Principal Findings

This study highlights the enablers of and impediments to trust in digital health technologies and services. Our results show that digital health encompasses a wide variety of health technology types and their respective services. Altogether, we identified 3 primary stakeholders: *patients*, *HCPs,* and *HAs*. Moreover, our findings map out cross-cutting *personal*, *technological*, and *institutional* trust elements in the form of enablers and impediments to trust in digital health technologies. Of these elements, sociodemographic factors and service provider reputation acted simultaneously as enablers and impediments.

A possible interpretation of the ambivalent nature of sociodemographic factors may lie in the fact that a lack of resources, be them material or educational, render people in a vulnerable state. Within health care settings, individuals often compensate for their vulnerability by perceiving health workers as potential threats [42]. The level of risk involved in instances of unfulfilled or broken trust impacts the willingness of vulnerable people to entrust individuals, institutions, or technologies with various tasks. In a similar fashion, those sitting at the high end of the socioeconomic spectrum may be prone to trust new technologies because of their perceived ability to control them. Alternatively, they may have higher expectations with regards to health care services and, thus, set the bar of trustworthiness much higher than the more disadvantaged strata of the population.

The ambiguity that we uncovered in this study reflects what other studies on trust vis-à-vis sociodemographic status have highlighted. Available evidence on the role of sociodemographic factors (eg, ethnicity, gender, and educational status) within the health care context is mixed. For instance, 1 study, has shown that patient characteristics (with the exception of age) rarely predict trust in patient-doctor relationships [43]. Conversely, others have identified patient characteristics such as age, ethnicity, income status, educational level, and literacy levels as crucial factors affecting the use of electronic health [20,44]. In light of these discrepant findings, further research is needed to clarify the underlying effects of sociodemographic factors in digital health.

A prevalent theme throughout this review was that stakeholders appear to trust profit-making entities such as insurance and pharmaceutical companies much less than they do public institutions like universities. This is a widespread phenomenon that reflects greater public assumptions about the private sector’s interests and profits [45]. Our findings support the importance of reputation to trust even though *service provider reputation* was identified as both a trust impediment and enabler. On the one hand, when a service provider embodies high ethical standards and is proficient at providing required services, they attain the advantage of shaping the expectations of stakeholders positively. In contrast, negative performance statistics of a service provider stand to give rise to negative expectations about their proficiency.

Despite stakeholder optimism about digital health tools, there are notable concerns about the accuracy of digital information exacerbated by the absence of uniform quality controls and standards [23]. Onora O’Neill has underscored the importance of enacting policies that address these challenges [26]. Based on the studies concerning Web-based health information included in this review, it was observed that patients and HCPs struggled to establish the quality of digital information. Consequently, in order to gauge the authenticity, veracity, and usefulness of digital health technologies or services, they relied quite significantly on *recommendations* from family members, colleagues, or acquaintances.

The FDA definition that we adopted for this review features personalized medicine as one of the components of digital health. Domains such as personalized medicine rely on the creation of large cohorts of deeply characterized individuals, as is the case with the 1 million participant research cohort being built for the Precision Medicine Initiative in the United States [3,46,47]. Success in this area will crucially depend on trust [48,49]. How to gain the degree of public support and personal commitment that is needed to build such infrastructures is far from obvious. In such cases, the ability to measure trustworthiness against a validated set of criteria will greatly increase the odds of success for such initiatives. Our study can be considered as a vital step in this direction, laying the conceptual groundwork for the development of such tools.

As we have shown, trust in digital health technologies and services depends on the interplay of a complex set of enablers and impediments. This study sheds light on what determines trust in digital health according to different stakeholders. More specifically, our findings can be of help in the implementation of innovative digital health technologies and services as well as in the management of existing digital health infrastructures. Building on insights from this study, actionable metrics such as the patient trust in telemedicine services tool can be developed to assess the trustworthiness of digital technologies in health care [50]. Each metric would need to undergo a validation process before being deployed in practice by HAs charged with monitoring or developing digital health services.

Overall, engaging with efforts to investigate the different dimensions of trust is particularly urgent given the growing attention from entities such as governments. This heightened level of attention is warranted due to the potential impacts of ever more innovative forms of digital health. Some approaches to digital health, in particular, those relying on big data, predictive analytics, and artificial intelligence [51-53] will require dedicated governance models in order to deliver on their promises while meeting the expectations of their users [54]. Reliable ways of measuring trustworthiness will, thus, be a key tool in such a rapidly evolving scenario.

### Limitations

A drawback to this study is the unequal number of studies in each digital health category. Although this was unlikely to have skewed our findings, there were relatively fewer studies on the newer forms of digital health such as wearable devices. Despite suggestions for reviews to be screened by 2 individuals, the volume and the complicated 2-step process involved in gleaning relevant information meant that only 1 author (AA) could fully screen all of the publications. Nevertheless, a second author (AB) screened 10% of the total publications for which a kappa statistic was calculated to ensure a minimal level of bias. Even though there was a moderate interrater agreement score (kappa=.661; 95% CI 0.465-0.857; *P*<.001), our kappa statistic is well above the .60 value that represents an inadequate agreement threshold [39]. Lastly, we acknowledge that scoping reviews can have several shortcomings [55]. However, the poorly-defined nature of both digital health and trust within the literature required a method that could map out the discourse and, thus, pave the way for a systematic review.

### Conclusion

Rapid advances in digital technologies and data science over the last few years are predicted to have a tangible impact on health care services, configuring a paradigm shift into what is now commonly referred to as digital health. Digital health, however, relies heavily on trust to succeed. What counts as the building blocks of trust in digital health systems has so far remained underexplored. In this study via a scoping review approach, we seek to fill this gap by analyzing what relevant stakeholders consider as the constitutive elements of trust in digital health. Overall, 278 qualitative, quantitative, mixed-methods, and intervention studies in English were included in this review. *Patients* and *HCPs* were the 2 most prominent stakeholders to trust, while *HAs* were a third and substantially less prominent stakeholder. Altogether, the trust elements that either enabled or hindered trust in digital health clustered into *personal*, *technological*, and *institutional* factors. This study paves the way for the implementation of the criteria necessary to measure and anticipate trust in emerging health care technologies.

## Appendix

Multimedia Appendix 1 Search queries.

Multimedia Appendix 2 Studies illustrating trust enablers and impediments.

Multimedia Appendix 3 List of study countries.

Multimedia Appendix 4 Health technology types.

## Abbreviations

EHR: electronic health record
FDA: Food and Drug Administration
HA: health administrator
HCP: health care professional
IT: information technology
mHealth: mobile health

## References

[1] Kostkova P (2015). Grand challenges in digital health. Front Public Health.

[2] (2018). World Health Organization.

[3] Vayena E, Haeusermann T, Adjekum A, Blasimme A (2018). Digital health: meeting the ethical and policy challenges. Swiss Med Wkly.

[4] Steinhubl SR, Topol EJ (2018). Digital medicine, on its way to being just plain medicine. npj Digital Med.

[5] Boogerd EA, Arts T, Engelen LJ, van de Belt TH (2015). “What Is eHealth”: Time for An Update?. JMIR Res Protoc.

[6] (2018). United States Food and Drug Administration.

[7] Duggal R, Brindle I, Bagenal J (2018). Digital healthcare: regulating the revolution. BMJ.

[8] Krenn L, Schlossman D (2017). Have Electronic Health Records Improved the Quality of Patient Care?. PM R.

[9] Hillestad R, Bigelow J, Bower A, Girosi F, Meili R, Scoville R, Taylor R (2005). Can electronic medical record systems transform health care? Potential health benefits, savings, and costs. Health Aff (Millwood).

[10] McKenna RM, Dwyer D, Rizzo JA (2017). Is HIT a hit? The impact of health information technology on inpatient hospital outcomes. Applied Economics.

[11] Agboola SO, Bates DW, Kvedar JC (2016). Digital Health and Patient Safety. JAMA.

[12] Horvitz E, Mulligan D (2015). Policy forum. Data, privacy, and the greater good. Science.

[13] Franko OI, Tirrell TF (2012). Smartphone app use among medical providers in ACGME training programs. J Med Syst.

[14] Steinhubl SR, Muse ED, Topol EJ (2015). The emerging field of mobile health. Sci Transl Med.

[15] Yusif S, Soar J, Hafeez-Baig A (2016). Older people, assistive technologies, and the barriers to adoption: A systematic review. Int J Med Inform.

[16] Mittelstadt B (2011). Ethical Issues of Personal Health Monitoring: A Literature Review.

[17] Chan M, Estève D, Fourniols J, Escriba C, Campo E (2012). Smart wearable systems: current status and future challenges. Artif Intell Med.

[18] Khoja S, Durrani H, Nayani P, Fahim A (2012). Scope of policy issues in eHealth: results from a structured literature review. J Med Internet Res.

[19] Strech D (2011). Ethical principles for physician rating sites. J Med Internet Res.

[20] O'Connor S, Hanlon P, O'Donnell CA, Garcia S, Glanville J, Mair FS (2016). Understanding factors affecting patient and public engagement and recruitment to digital health interventions: a systematic review of qualitative studies. BMC Med Inform Decis Mak.

[21] Torous J, Roberts LW (2017). Needed Innovation in Digital Health and Smartphone Applications for Mental Health: Transparency and Trust. JAMA Psychiatry.

[22] Yamin CK, Emani S, Williams DH, Lipsitz SR, Karson AS, Wald JS, Bates DW (2011). The digital divide in adoption and use of a personal health record. Arch Intern Med.

[23] Kelton K, Fleischmann KR, Wallace WA (2008). Trust in digital information. J. Am. Soc. Inf. Sci.

[24] Adjekum A, Ienca M, Vayena E (2017). What Is Trust? Ethics and Risk Governance in Precision Medicine and Predictive Analytics. OMICS.

[25] O'Neill O (2002). A Question of Trust : The BBC Reith Lectures 2002.

[26] O'Neill O (2017). Intelligent Trust in a Digital World. New Perspectives Quarterly.

[27] Dorr GS, Lipkin M (1999). The doctor-patient relationship: challenges, opportunities, and strategies. J Gen Intern Med.

[28] Bending ZJ (2015). Reconceptualising the doctor-patient relationship: recognising the role of trust in contemporary health care. J Bioeth Inq.

[29] O'Neill O (2002). Autonomy and Trust in Bioethics.

[30] Gilson L (2003). Trust and the development of health care as a social institution. Soc Sci Med.

[31] Rowe R, Calnan M (2006). Trust relations in health care: developing a theoretical framework for the “new” NHS. J Health Organ Manag.

[32] Hawley K (2012). Trust, Distrust and Commitment. Noûs.

[33] Sztompka P (1999). Trust: a Sociological Theory.

[34] Giddens A, Anthony (1990). The consequences of modernity.

[35] Montague EN, Kleiner BM, Winchester WW (2009). Empirically understanding trust in medical technology. International Journal of Industrial Ergonomics.

[36] Arksey H, O'Malley L (2005). Scoping studies: towards a methodological framework. International Journal of Social Research Methodology.

[37] Tung F, Chang S, Chou C (2008). An extension of trust and TAM model with IDT in the adoption of the electronic logistics information system in HIS in the medical industry. Int J Med Inform.

[38] Buscemi N, Hartling L, Vandermeer B, Tjosvold L, Klassen TP (2006). Single data extraction generated more errors than double data extraction in systematic reviews. J Clin Epidemiol.

[39] McHugh ML (2012). Interrater reliability: the kappa statistic. Biochem Med (Zagreb).

[40] Moher D, Liberati A, Tetzlaff J, Altman DG (2009). Preferred reporting items for systematic reviews and meta-analyses: the PRISMA statement. BMJ.

[41] Davis FD (1989). Perceived Usefulness, Perceived Ease of Use, and User Acceptance of Information Technology. MIS Quarterly.

[42] Plomp HN, Ballast N (2010). Trust and vulnerability in doctor-patient relations in occupational health. Occup Med (Lond).

[43] Hall MA, Dugan E, Zheng B, Mishra AK (2001). Trust in physicians and medical institutions: what is it, can it be measured, and does it matter?. Milbank Q.

[44] Hardiker NR, Grant MJ (2011). Factors that influence public engagement with eHealth: A literature review. Int J Med Inform.

[45] Abelson J, Miller FA, Giacomini M (2009). What does it mean to trust a health system? A qualitative study of Canadian health care values. Health Policy.

[46] Collins FS, Varmus H (2015). A new initiative on precision medicine. N Engl J Med.

[47] Fogel AL, Kvedar JC (2018). Artificial intelligence powers digital medicine. Digital Med.

[48] Blasimme A, Vayena E (2016). Becoming partners, retaining autonomy: ethical considerations on the development of precision medicine. BMC Med Ethics.

[49] Blasimme A, Vayena E (2016). “Tailored-to-You”: Public Engagement and the Political Legitimation of Precision Medicine. Perspectives in Biology and Medicine.

[50] Velsen LV, Tabak M, Hermens H (2017). Measuring patient trust in telemedicine services: Development of a survey instrument and its validation for an anticoagulation web-service. Int J Med Inform.

[51] Chen JH, Asch SM (2017). Machine Learning and Prediction in Medicine - Beyond the Peak of Inflated Expectations. N Engl J Med.

[52] Shapshay SM (2014). Artificial intelligence: the future of medicine?. JAMA Otolaryngol Head Neck Surg.

[53] Beam AL, Kohane IS (2016). Translating Artificial Intelligence Into Clinical Care. JAMA.

[54] Vayena E, Blasimme A (2018). Health Research with Big Data: Time for Systemic Oversight. J Law Med Ethics.

[55] Grant MJ, Booth A (2009). A typology of reviews: an analysis of 14 review types and associated methodologies. Health Info Libr J.

