# Supplementary material for: Elements of Trust in Digital Health Systems: Scoping Review
Source: J Med Internet Res. 2018 Dec 13;20(12):e11254. doi: 10.2196/11254 (PMC6315261; doi:10.2196/11254)
Supplement: Multimedia Appendix 1 [file jmir_v20i12e11254_app1.pdf]

Multimedia Appendix 1 - Search Queries

Cinahl:

(MH “Perception+”) OR(MH “Attitude+”) OR TI (perception* OR perceiv* OR expect* OR prospect OR prospects OR perspective* OR experienc* OR attitude) OR AB (perception* OR perceiv* OR expect* OR prospect OR prospects OR perspective* OR exprienc* OR attitude)

(MH “Electronic Health Records”) OR (MH “Telehealth+”) OR (MH “Mobile Applications”) OR TX (((digital or electronic* OR virtual) W3 (medicine OR health*)) OR e-health * OR e-medicine OR (tele-health* OR tele-medicine OR telehealth* OR telemedicine) OR ((precision OR personalized medicine OR individualized) W3 medicine) OR (electronic W3 (health OR medical OR patient) W3 record*) OR (mobile W3 health*) OR m-health* OR mhealth* OR ((health OR medical) W3 (app or apps OR application*)) OR (((mobile OR cell*) W3 (device* OR phone*)) OR smartphone* OR iphone OR ipad OR tablet OR wearable*) AND (medic* OR health*)) OR (((MH “Computers, Hand-Held”)) AND (TI(medicine OR health*) OR AB(medicine OR health*)))

(MH “Trust”) OR (MH “Confidence”) OR TI (trust* OR mistrust* OR distrust* OR credibility OR (confidence NOT (confidence N3 interval*)) OR reliance) OR AB (trust* OR mistrust* OR distrust* OR credibility OR (confidence NOT (confidence N3 interval*)) OR reliance)

Medline:

(MH “Perception+”) OR (MH “Attitude+”) OR TI (perception* OR perceiv* OR expect* OR prospect OR prospects OR perspective* OR experienc* OR attitude) OR AB (perception* OR perceiv* OR expect* OR prospect OR prospects OR perspective* OR experienc* OR attitude)

(MH “Precision Medicine”) OR (MH “Telemedicine+”) OR (MH “Electronic Health Records+”) OR (MH “Mobile Applications”) OR TX (((digital OR electronic* OR virtual) W3 (medicine OR health*)) OR e-health* OR e-medicine OR (tele-health* OR tele-medicine OR telehealth* OR telemedicine) OR ((precision OR personalized OR individualized) W3 medicine) OR (electronic W3 (health OR medical OR patient) W3 record*) OR (mobile W3 health*) OR m-health* OR mhealth* OR ((health OR medical) W3 (app OR apps or application*)) OR (((mobile OR cell*) W3 (device* OR phone*)) OR smartphone*OR iphone OR ipad OR tablet OR wearable*) AND (medic* OR health*)) OR ((MH “Computers, Handheld+”) AND (TI(medicine OR health*) OR AB(medicine OR health*)))

(MH “Trust”) OR TI (trust* OR mistrust* OR distrust* OR credibility OR (confidence NOT (confidence N3 interval*)) OR reliance) OR AB (trust* OR mistrust* OR distrust* OR credibility OR (confidence NOT (confidence N3 interval*)) OR reliance)

PsycInfo:

(((((DE “Perception”) OR (DE “Expectations”)) OR (DE “Experiences (Events)”)) OR (DE “Life Experiences”))) AND (DE “Attitudes” OR DE “Abortion (Attitudes Toward)” OR “Adolescents Attitudes” OR DE “Adult Attitudes” OR DE “Aged (Attitudes Toward)” OR DE “Aging (Attitudes Toward)” OR DE “Child Attitudes” OR DE “Childrearing Attitudes” OR DE “Client Attitudes” OR DE “Community Attitudes” OR DE “Computer Attitudes” OR DE “Consumer Attitudes” OR DE “Counselor Attitudes” OR DE “Death Attitudes” OR DE “Disabled (Attitudes Toward)” OR DE “Drug Usage Attitudes” OR DE “Eating Attitudes” OR DE “Employee Attitudes” OR DE “Employer Attitudes” OR DE “Environmental Attitudes” OR DE “Explicit Attitudes” OR DE “Family Planning Attitudes” OR DE “Female Attitudes” OR DE “Health Attitudes” OR DE “Health Personnel Attitudes” OR DE “Homosexuality (Attitudes Toward)” OR DE “Implicit Attitudes” OR DE “Job Applicant Attitudes” OR DE “Male Attitudes” OR DE “Marriage Attitudes” OR DE “obesity (Attitudes Toward)” OR DE “Occupational Attitudes” OR DE “Parental Attitudes” OR DE “Paternalism” OR DE “Physical Illness (Attitudes Toward)” OR DE “Political Attitudes” Or DE “Psychologist Attitudes” OR DE “Public Opinion” OR DE “Racial and Ethnic Attitudes” OR DE “Sex Role Attitudes” OR DE “Sexual Attitudes” OR DE “Socioeconomic Class Attitudes” OR DE “Sports (Attitudes Toward)” OR DE “Stereotyped Attitudes” OR DE “Student Attitudes” OR DE “Teacher Attitudes” OR DE “Work (Attitudes Toward)” OR DE “Prejudice”) OR TI (perception* OR perceiv* OR expect* OR prospect OR prospects OR perspective* OR experienc* OR attitude) OR AB (perception* OR perceiv* OR expect* OR prospect OR prospects OR perspective* OR experienc* OR attitude)

((DE “Online Therapy”) OR (DE “Telemedicine”)) OR TX (((digital OR electronic* OR virtual) W3 (medicine OR health*)) OR e-health* OR e-medicine OR tele-health* OR tele-medicine OR telehealth* OR telemedicine*) OR ((precision OR personalized OR individualized) W3 (health OR medical OR patient) W3 record*) OR (mobile W3 health*) OR m-health* OR mhealth* OR ((health OR medical) W3 (app OR apps OR application*)) OR (((mobile OR cell*) W3 (device* OR phone*)) OR smarthone* OR iphone OR ipad OR tablet OR wearable*) AND (medic* OR health*)) OR (((DE “Mobile Devices”)) AND (TI(medicine OR health*) OR AB(medicine OR health*)))

((DE “Trust (Social Behavior)”) OR (DE “Suspicion”)) OR (DE “Credibility”) OR TI (trust* OR mistrust* OR distrust* OR credibility OR (confidence NOT (confidence N3 interval*)) OR reliance) OR AB (trust* OR mistrust* OR distrust* OR credibility OR (confidence NOT (confidence N3 interval*)) OR reliance)

Web of Science:

TS=(perception* OR perceiv* OR expect* OR prospect OR prospects OR perspective* OR experienc* OR attitude)

TS=(((digital OR electronic* OR virtual) NEAR/3 (medicine OR health*)) OR e-health* OR e-medicine OR tele-health* OR tele-medicine OR telehealth* OR telemedicine OR ((precision OR personalized OR individualized) NEAR/3 medicine) OR (electronic NEAR/3 (health OR medical OR patient) NEAR/3 record*) OR (mobile NEAR/3 health*) OR m-health* OR mhealth* OR ((health OR medical) NESR/3 (app OR apps OR application*)) OR (((mobile OR cell** NEAR/3 (device* OR phone*)) OR smartphone* OR iphone OR ipad OR tablet OR wearable*) AND (medic* OR health*)))

TS= (trust* OR mistrust* OR distrust OR leeriness OR suspicious* OR credibility OR (confidence NOT (confidence NEAR/3 interval*)) OR reliance)

Embase:

‘perception’/exp OR ‘expectation’/exp OR ‘experience’/exp OR ‘attitude’/exp OR perception*:ti,ab OR perceiv*:ti,ab OR expect*:ti,ab OR prospect:ti,ab OR prospects:ti,ab OR perspective*ti,ab OR experienc*:ti,ab OR attitude:ti,ab

‘personalized medicine’/exp OR ‘telehealth’/exp OR ‘mobile application’exp OR ‘electronic medical record’/exp OR (((digital OR electronic* OR virtual) NEXT/3 (medicine OR health*)):ti,ab) OR ‘e health*’:ti,ab OR ‘e medicine’:ti,ab OR ‘tele health*:ti,ab OR ‘tele medicine’:ti,ab OR telehealth*:ti,ab OR telemedicine:ti,ab OR (((precision OR personalized OR individualized) NEXT/3 medicine):ti,ab OR ((electronic NEXT/3 (health OR medical OR patient) NEXT/3 record*):ti,ab) OR((mobile NEXT/3 health*):ti,ab) OR ‘, health*:ti,ab R mhealth*:ti,ab OR (((health OR medical) NEXT/3 (app OR apps OR application*)):ti,ab) OR (((((mobile OR cell*) NEXT/3 (device* OR phone*)):ti,ab) OR smartphone*:ti,ab OR iphone:ti,ab OR ipad:ti,ab OR tablet:ti,ab OR wearable*:ti,ab) AND (medic*:ti,ab OR health*:ti,ab)) OR (‘mobile phone’/exp AND (medicine:ti,ab OR health*:ti,ab))

‘trust’/exp OR trust*:ti,ab OR mistrust*:ti,ab OR distrust*:ti,ab OR leeriness:ti,ab OR suspicious*:ti,ab OR credibility:ti,ab OR (confidence:ti,ab NOT (confidence NEXT/3 interval*):ti,ab) OR reliance:ti,ab
