# Supplementary material for: Elements of Trust in Digital Health Systems: Scoping Review
Source: J Med Internet Res. 2018 Dec 13;20(12):e11254. doi: 10.2196/11254 (PMC6315261; doi:10.2196/11254)
Supplement: Multimedia Appendix 2 [file jmir_v20i12e11254_app2.pdf]

## Appendix 3: Studies illustrating trust enablers and impediments

| Trust Enablers & Impediments | Corresponding Publications |
|---|---|
| Altruism | [1-9] |
| Poor information quality | [10-60] |
| Customizable design features | [15, 23, 24, 28, 31, 34, 41, 49, 52, 60-78] |
| Decreased workloads | [4, 6-11, 13-15, 20, 21, 24, 27, 29, 31, 36, 39, 45-48, 51, 55, 56, 59, 62, 73, 76, 79-131] |
| Ease of use | [13, 15, 20, 22, 25, 26, 28, 36, 39, 54, 56, 57, 63, 68, 70, 71, 73, 75, 76, 78, 85, 89, 92, 97, 103, 110, 112, 116, 132-156] |
| Self-efficacy | [61, 118, 145, 157-168] |
| Fair data access | [5, 6, 8, 9, 12, 40, 72, 87, 95, 104, 128, 166, 169-177] |
| Fear of data exploitation | [1, 4, 5, 7, 9, 40, 55, 60, 72, 95, 115, 116, 119, 167, 171, 173, 174, 178-185] |
| Guidelines for standardized use | [9, 16, 19, 20, 62, 73, 81, 83, 85, 92, 99, 119, 121, 122, 131, 175, 180, 182, 186-189] |
| Improved communication | [11, 12, 14, 20, 23, 25, 30, 40, 42, 47, 60, 64, 68, 69, 75, 76, 84, 88, 101, 106, 109, 116, 117, 120, 121, 123, 125, 132, 135, 137, 140, 141, 147, 155, 166, 177, 185, 190-198] |
| Limited accessibility | [4, 8, 11, 15, 36, 42, 57, 62, 64, 70, 72, 73, 80, 81, 85, 86, 89, 92-94, 96, 104, 110-112, 115-117, 119, 123, 125, 130-132, 135, 137-139, 147, 151, 155, 172, 174, 179, 181, 185, 189, 197, 199-205] |
| Inadequate publicity | [6, 7, 16, 19, 35, 41, 60, 66, 72, 81, 82, 87, 93, 94, 104, 108, 110-112, 115, 118, 119, 131, 134, 140, 151, 158, 164, 172, 182, 185, 187, 188, 194, 206-215] |
| Initial face-to-face contact | [13, 20, 24, 57, 60, 70, 77, 85, 88, 93, 98, 102, 105, 106, 110, 112, 117, 121, 131, 135, 137, 140, 150, 177, 184, 191, 193, 205, 206, 216-226] |
| Insufficient training | [10, 13, 15, 19, 20, 27, 29, 42, 45, 51, 55, 56, 58, 59, 61, 64, 68, 71, 73, 74, 76, 77, 79, 83, 86, 87, 89-92, 98, 111, 114, 118, 120, 122, 126, 135, 136, 182, 187, 189, 200, 209, 213, 214, 227-234] |
| Interoperability | [15, 20, 24, 62, 66, 78, 81, 122, 131, 235] |
| Privacy | [1, 2, 6, 7, 9, 13, 23-25, 29, 33, 40, 42, 46, 48, 50, 54, 57, 60, 67, 70, 76, 77, 82, 84, 89, 92, 95, 104-106, 110, 115, 116, 122, 123, 130, 131, 134, 137, 139, 166, 171, 172, 175-177, 179-181, 183, 185, 189, 190, 204, 212, 218, 219, 222-224, 232, 236-246] |
| Recommendation by others | [24, 26, 44, 64, 66, 72, 87, 88, 135, 154, 168, 184, 231, 235, 247-249] |
| Time consuming | [10, 12, 14-16, 21, 22, 29, 31, 34, 39, 40, 42, 43, 46-48, 55, 59, 62, 72, 79, 82, 83, 85, 86, 88, 91, 106, 109, 114, 119, 133, 140, 141, 187, 193, 201, 222, 226, 250, 251] |

| | |
|---|---|
| Service provider reputation | [1, 4-9, 13, 17, 23, 26, 28, 33, 43, 44, 46, 47, 52, 55, 57, 58, 60, 63, 65, 73, 86, 108, 115, 119, 127, 145, 146, 148, 156, 161, 164-166, 170, 173, 174, 180, 183-185, 187, 188, 192, 203, 219, 224, 225, 231, 238, 240, 242, 247, 251-264] |
| Socio-demographic factors | [1, 4, 9, 11, 18, 22, 30, 32, 33, 41, 43, 44, 65, 67, 72, 75, 81, 82, 87, 88, 93, 100, 104, 105, 108, 112, 115, 116, 132, 140, 141, 148, 150, 151, 153, 154, 157, 158, 161, 162, 165, 168, 172, 174, 179-181, 185, 188, 189, 192, 194, 195, 197, 199, 202, 203, 205, 208, 210, 213, 216, 224, 228, 231, 233, 238, 239, 241, 243, 244, 246, 251, 253, 254, 259, 264-271] |
| Stakeholder engagement | [1, 2, 4, 5, 9, 12, 13, 15, 41, 42, 48, 55, 58, 60, 61, 65, 70, 73, 74, 79, 80, 83, 88, 93, 94, 96-98, 102, 108, 114, 115, 118, 119, 128, 129, 131, 135, 140, 142, 153, 154, 170, 181, 183, 187, 188, 190, 193, 196, 198, 201, 206, 208, 210, 211, 216, 218, 227, 230, 232, 244, 250, 251, 255, 256, 259, 264, 267, 272, 273] |
| Excessive costs | [8, 11, 15, 42, 64-66, 73, 77, 80, 81, 92, 93, 104, 110, 111, 115, 119, 131, 137, 139, 147, 172, 174, 179, 189, 197, 200, 201, 203-206, 270] |
| Defective technology | [15, 21, 29, 33, 47, 51, 55, 64, 70, 74, 76, 79, 83, 85, 86, 93, 97, 98, 105, 106, 111, 114, 125, 137, 156, 169, 193, 219, 223, 233, 251, 274] |
| Usefulness | [2, 4, 11, 13, 16, 17, 20, 23, 24, 26, 32, 33, 37, 42, 51, 54, 55, 57-61, 63, 66, 68, 69, 72, 77-79, 87, 88, 90, 94, 96, 100, 104, 105, 107, 110-113, 122, 125, 132, 134, 137-141, 143, 144, 146, 149-154, 156-158, 160, 163, 164, 169, 177, 180, 185, 186, 188, 190-193, 195, 197, 202, 203, 205, 206, 210, 212, 214, 218, 221, 223, 226, 229, 232, 233, 236, 239, 240, 245, 255-257, 260, 262, 263, 265, 266, 272, 275-278] |


1. Buseh AG, Stevens PE, Millon-Underwood S, Townsend L, Kelber ST. Community leaders' perspectives on engaging African Americans in biobanks and other human genetics initiatives. Journal Of Community Genetics. 2013;4(4):483-94. PMID: 23813337. doi: 10.1007/s12687-013-0155-z.
2. Coulson NS, Shaw RL. Nurturing health-related online support groups: Exploring the experiences of patient moderators. Computers in Human Behavior. 2013;29(4):1695-701. doi: 10.1016/j.chb.2013.02.003.
3. Grant A, Ure J, Nicolson DJ, Hanley J, Sheikh A, McKinstry B, et al. Acceptability and perceived barriers and facilitators to creating a national research register to enable 'direct to patient' enrolment into research: the Scottish Health Research Register (SHARE). BMC Health Services Research. 2013;13:422. PMID: 24139174. doi: 10.1186/1472-6963-13-422.
4. Halbert CH, McDonald J, Vadaparampil S, Rice L, Jefferson M. Conducting Precision Medicine Research with African Americans. Plos One. 2016;11(7):e0154850-e. PMID: 27441706. doi: 10.1371/journal.pone.0154850.

5. Overby CL, Maloney KA, Alestock TD, Chavez J, Berman D, Sharaf RM, et al. Prioritizing Approaches to Engage Community Members and Build Trust in Biobanks: A Survey of Attitudes and Opinions of Adults within Outpatient Practices at the University of Maryland. Journal Of Personalized Medicine. 2015;5(3):264-79. PMID: 26226006. doi: 10.3390/jpm5030264.
6. Platt J, Kardia S. Public trust in health information sharing: implications for biobanking and electronic health record systems. Journal Of Personalized Medicine. 2015;5(1):3-21. PMID: 25654300. doi: 10.3390/jpm5010003.
7. Sanderson SC, Brothers KB, Mercaldo ND, Clayton EW, Antommaria AHM, Aufox SA, et al. Public Attitudes toward Consent and Data Sharing in Biobank Research: A Large Multi-site Experimental Survey in the US. American Journal Of Human Genetics. 2017;100(3):414-27. PMID: 28190457. doi: 10.1016/j.ajhg.2017.01.021.
8. Su Y, Borry P, Otte IC, Howard HC. "It's our DNA, we deserve the right to test!" A content analysis of a petition for the right to access direct-to-consumer genetic testing. Personalized Medicine. 2013;10(7):729-39. doi: 10.2217/pme.13.69.
9. Trinidad SB, Fullerton SM, Bares JM, Jarvik GP, Larson EB, Burke W. Genomic research and wide data sharing: Views of prospective participants. Genetics in Medicine. 2010;12(8):486-95. doi: 10.1097/GIM.0b013e3181e38f9e.
10. Adams A, Adams R, Thorogood M, Buckingham C. Barriers to the use of e-health technology in nurse practitioner-patient consultations. Informatics In Primary Care. 2007;15(2):103-9. PMID: 17877872.
11. Andreassen HK, Bujnowska-Fedak MM, Chronaki CE, Dumitru RC, Pudule I, Santana S, et al. European citizens' use of E-health services: A study of seven countries. Bmc Public Health. 2007 Apr;7:7. doi: 10.1186/1471-2458-7-53.
12. Asan O, Tyszka J, Fletcher KE. Capturing the patients' voices: Planning for patient-centered electronic health record use. International Journal Of Medical Informatics. 2016;95:1-7. PMID: 27697228. doi: 10.1016/j.ijmedinf.2016.08.002.
13. Best P, Manktelow R, Taylor BJ. Social Work and Social Media: Online Help-Seeking and the Mental Well-Being of Adolescent Males. British Journal of Social Work. 2016;46(1):257-76. PMID: 112544086. doi: 10.1093/bjsw/bcu130.
14. Chase DA, Ash JS, Cohen DJ, Hall J, Olson GM, Dorr DA. The EHR's roles in collaboration between providers: A qualitative study. AMIA Annual Symposium Proceedings AMIA Symposium. 2014;2014:1718-27. PMID: 25954444.
15. Cranfield S, Hendy J, Reeves B, Hutchings A, Collin S, Fulop N. Investigating healthcare IT innovations: a "conceptual blending" approach. J Health Organ Manag. 2015;29(7):1131-48. doi: 10.1108/jhom-08-2015-0121.
16. Crosson JC, Schueth AJ, Isaacson N, Bell DS. Early adopters of electronic prescribing struggle to make meaningful use of formulary checks and medication history documentation. Journal Of The American Board Of Family Medicine: JABFM. 2012;25(1):24-32. PMID: 22218621. doi: 10.3122/jabfm.2012.01.100297.
17. Dahri K, Gong Y, Loewen P. A quantitative and qualitative assessment of the utilization of mobile computing devices by clinical pharmacists. Health Policy and Technology. 2016;5(3):285-90. doi: 10.1016/j.hlpt.2016.03.007.
18. Deng Z, Liu S, Hinz O. The health information seeking and usage behavior intention of Chinese consumers through mobile phones. Information Technology & People. 2015;28(2):405-23. doi: 10.1108/ITP-03-2014-0053.

19. Devitt N, Murphy J. A survey of the information management and technology training needs of doctors in an acute NHS trust in the United Kingdom. Health Information And Libraries Journal. 2004;21(3):164-72. PMID: 15318914.
20. Embi PJ, Weir C, Efthimiadis EN, Thielke SM, Hedeen AN, Hammond KW. Computerized provider documentation: findings and implications of a multisite study of clinicians and administrators. Journal Of The American Medical Informatics Association: JAMIA. 2013;20(4):718-26. PMID: 23355462. doi: 10.1136/amiajnl-2012-000946.
21. Ernesäter A, Holmström I, Engström M. Telenurses' experiences of working with computerized decision support: supporting, inhibiting and quality improving. Journal of Advanced Nursing. 2009;65(5):1074-83. doi: 10.1111/j.1365-2648.2009.04966.x.
22. Escoffery C, Miner KR, Adame DD, Butler S, McCormick L, Mendell E. Internet use for health information among college students. Journal of American College Health. 2005;53(4):183-8. PMID: 15663067
23. Glynn L, Casey M, Walsh J, Hayes PS, Harte RP, Heaney D. Patients' views and experiences of technology based self-management tools for the treatment of hypertension in the community: A qualitative study. BMC Family Practice. 2015;16:119-. PMID: 26354752. doi: 10.1186/s12875-015-0333-7.
24. Goetz M, Muller M, Matthies LM, Hansen J, Doster A, Szabo A, et al. Perceptions of Patient Engagement Applications During Pregnancy: A Qualitative Assessment of the Patient's Perspective. Jmir Mhealth and Uhealth. 2017 May;5(5):12. PMID: 28550005. doi: 10.2196/mhealth.7040.
25. Haggstrom DA, Saleem JJ, Russ AL, Jones J, Russell SA, Chumbler NR. Lessons learned from usability testing of the VA's personal health record. Journal Of The American Medical Informatics Association: JAMIA. 2011;18 Suppl 1:i13-i7. PMID: 21984604. doi: 10.1136/amiajnl-2010-000082.
26. Hajli MN, Sims J, Featherman M, Love PED. Credibility of information in online communities. Journal of Strategic Marketing. 2015;23(3):238-53. doi: 10.1080/0965254X.2014.920904.
27. Hanssen H, Norheim A, Hanson E. How can web-based training facilitate a more carer friendly practice in community-based health and social care services in Norway? Staff experiences and implementation challenges. Health & Social Care in the Community. 2017;25(2):559-68. doi: 10.1111/hsc.12343.
28. Harris PR, Sillence E, Briggs P. Perceived Threat and Corroboration: Key Factors That Improve a Predictive Model of Trust in Internet-based Health Information and Advice. Journal of Medical Internet Research. 2011 Jul-Sep;13(3):13. doi: 10.2196/jmir.1821.
29. Hart A, Henwood F, Jones A. Views of heads of midwifery on electronic patient records. British Journal of Midwifery. 2003;11(1):53-7. doi: 10.12968/bjom.2003.11.1.11016
30. Hesse BW, Nelson DE, Kreps GL, Croyle RT, Arora NK, Rimer BK, et al. Trust and sources of health information: the impact of the Internet and its implications for health care providers: findings from the first Health Information National Trends Survey. Archives Of Internal Medicine. 2005;165(22):2618-24. PMID: 16344419.
31. Hoff T. Deskilling and adaptation among primary care physicians using two work innovations. Health Care Management Review. 2011;36(4):338-48. PMID: 21685794. doi: 10.1097/HMR.0b013e31821826a1.
32. Hu X, Bell RA, Kravitz RL, Orrange S. The prepared patient: Information seeking of online support group members before their medical appointments. Journal of Health Communication. 2012;17(8):960-78. doi: 10.1080/10810730.2011.650828.

33. Illiger K, Hupka M, von Jan U, Wichelhaus D, Albrecht U-V. Mobile technologies: expectancy, usage, and acceptance of clinical staff and patients at a university medical center. JMIR Mhealth And Uhealth. 2014;2(4):e42-e. PMID: 25338094. doi: 10.2196/mhealth.3799.
34. Jensen LG, Bossen C. Factors affecting physicians' use of a dedicated overview interface in an electronic health record: The importance of standard information and standard documentation. International Journal Of Medical Informatics. 2016;87:44-53. PMID: 26806711. doi: 10.1016/j.ijmedinf.2015.12.009.
35. Jiménez-Pernett J, de Labry-Lima AO, Bermúdez-Tamayo C, García-Gutiérrez JF, del Carmen Salcedo-Sánchez M. Use of the internet as a source of health information by Spanish adolescents. BMC Medical Informatics And Decision Making. 2010;10:6-. PMID: 20113486. doi: 10.1186/1472-6947-10-6.
36. Johansson P, Petersson G, Saveman B-I, Nilsson G. Experience of mobile devices in nursing practice. Nordic Journal of Nursing Research & Clinical Studies / Vård i Norden. 2012;32(4):50-4.
37. Johansson PE, Petersson GI, Nilsson GC. Nursing students' experience of using a personal digital assistant (PDA) in clinical practice - an intervention study. Nurse Education Today. 2013;33(10):1246-51. PMID: 22999410. PMID: 21988433. doi: 10.1016/j.nedt.2012.08.019.
38. King J, Patel V, Jamoom EW, Furukawa MF. Clinical benefits of electronic health record use: National findings. Health Services Research. 2014;49(1 PART 2):392-404. doi: 10.1111/1475-6773.12135.
39. Koskela T, Sandstrom S, Makinen J, Liira H. User perspectives on an electronic decision-support tool performing comprehensive medication reviews - a focus group study with physicians and nurses. Bmc Medical Informatics and Decision Making. 2016 Jan;16:9. doi: 10.1186/s12911-016-0245-z.
40. Lehnbom EC, McLachlan A, Brien J-AE. A qualitative study of Australians' opinions about personally controlled electronic health records. Studies In Health Technology And Informatics. 2012;178:105-10. PMID: 22797027.
41. Manafò E, Wong S. Promoting eHealth literacy in older adults: key informant perspectives. Canadian Journal Of Dietetic Practice And Research: A Publication Of Dietitians Of Canada = Revue Canadienne De La Pratique Et De La Recherche En Dietetique: Une Publication Des Dietetistes Du Canada. 2013;74(1):37-41. PMID: 23449214.
42. Mannan R, Murphy J, Jones M. Is primary care ready to embrace e-health? A qualitative study of staff in a London primary care trust. Informatics In Primary Care. 2006;14(2):121-31. PMID: 17059701.
43. Marrie RA, Salter AR, Tyry T, Fox RJ, Cutter GR. Preferred sources of health information in persons with multiple sclerosis: degree of trust and information sought. Journal Of Medical Internet Research. 2013;15(4):e67-e. PMID: 23635393. doi: 10.2196/jmir.2466.
44. McPherson AC, Gofine ML, Stinson J. Seeing is believing? A mixed-methods study exploring the quality and perceived trustworthiness of online information about chronic conditions aimed at children and young people. Health Communication. 2014;29(5):473-82. doi: 10.1080/10410236.2013.768325.
45. Meum T, Wangensteen G, Soleng KS, Wynn R. How does nursing staff perceive the use of electronic handover reports? A questionnaire-based study. International Journal Of

Telemedicine And Applications. 2011;2011:505426-. PMID: 21760779. doi: 10.1155/2011/505426.
46. Morin D, Tourigny A, Pelletier D, Robichaud L, Mathieu L, Vézina A, et al. Seniors' views on the use of electronic health records. Informatics In Primary Care. 2005;13(2):125-33. PMID: 15992497.
47. Nerminathan A, Harrison A, Phelps M, Scott KM, Alexander S. Doctors' use of mobile devices in the clinical setting: a mixed methods study. Internal Medicine Journal. 2017;47(3):291-8. PMID: 27925381. doi: 10.1111/imj.13349.
48. Novek J, Bettess S, Burke K, Johnston P. Nurses' perceptions of the reliability of an automated medication dispensing system. Journal of Nursing Care Quality. 2000;14(2):1-13. PMID: 10646296.
49. Pang PCI, Chang S, Verspoor K, Pearce J. Designing Health Websites Based on Users' Web-Based Information-Seeking Behaviors: A Mixed-Method Observational Study. Journal of Medical Internet Research. 2016 Jun;18(6):15. doi: 10.2196/jmir.5661.
50. Qiao Y, Asan O, Montague E. Factors associated with patient trust in electronic health records used in primary care settings. Health Policy and Technology. 2015;4(4):357-63. doi: 10.1016/j.hlpt.2015.08.001.
51. Ricks E, Benjamin V, Williams M. Experiences of registered nurses with regard to accessing health information at the point-of-care via mobile computing devices. Curationis. 2015;38(2):1498-. PMID: 26842084. doi: 10.4102/curationis.v38i2.1498.
52. Sanders K, Valle MS, Vinaras M, Llorente C. Do we trust and are we empowered by "Dr. Google"? Older Spaniards' uses and views of digital healthcare communication. Public Relat Rev. 2015 Dec;41(5):794-800. doi: 10.1016/j.pubrev.2015.06.015.
53. Sarkar U, Gourley GI, Lyles CR, Tieu L, Clarity C, Newmark L, et al. Usability of Commercially Available Mobile Applications for Diverse Patients. Journal Of General Internal Medicine. 2016;31(12):1417-26. PMID: 27418347.
54. Schnall R, Higgins T, Brown W, Carballo-Dieguez A, Bakken S. Trust, Perceived Risk, Perceived Ease of Use and Perceived Usefulness as Factors Related to mHealth Technology Use. Studies In Health Technology And Informatics. 2015;216:467-71. PMID: 26262094.
55. Shield RR, Goldman RE, Anthony DA, Wang N, Doyle RJ, Borkan J. Gradual electronic health record implementation: new insights on physician and patient adaptation. Annals Of Family Medicine. 2010;8(4):316-26. PMID: 20644186. doi: 10.1370/afm.1136.
56. Terry AL, Brown JB, Bestard Denomme L, Thind A, Stewart M. Perspectives on electronic medical record implementation after two years of use in primary health care practice. Journal Of The American Board Of Family Medicine: JABFM. 2012;25(4):522-7. PMID: 22773720. doi: 10.3122/jabfm.2012.04.110089.
57. Thompson MJ, Valdez RS. Online Filipino-Americans' perspectives on informatics-enabled health management. Health Policy and Technology. 2015;4(4):320-36. doi: 10.1016/j.hlpt.2015.08.004.
58. Townsend A, Leese J, Adam P, McDonald M, Li LC, Kerr S, et al. eHealth, Participatory Medicine, and Ethical Care: A Focus Group Study of Patients' and Health Care Providers' Use of Health-Related Internet Information. Journal of medical Internet research. 2015;17(6):e155. PMID: 26099267. doi: 10.2196/jmir.3792.
59. van Kerkhof LWM, van der Laar CWE, de Jong C, Weda M, Hegger I. Characterization of Apps and Other e-Tools for Medication Use: Insights Into Possible Benefits and Risks. JMIR Mhealth And Uhealth. 2016;4(2):e34-e. PMID: 27052946. doi: 10.2196/mhealth.4149.

60. Van Velsen L, Wildevuur S, Flierman I, Van Schooten B, Tabak M, Hermens H. Trust in telemedicine portals for rehabilitation care: an exploratory focus group study with patients and healthcare professionals. BMC Medical Informatics And Decision Making. 2016;16:11-. PMID: 26818611. doi: 10.1186/s12911-016-0250-2.
61. Chu A, Huber J, Mastel-Smith B, Cesario S. 'Partnering with seniors for better health': computer use and internet health information retrieval among older adults in a low socioeconomic community. Journal of the Medical Library Association. 2009;97(1):12-20. PMID: 105632239. PMID: 19159002. doi: 10.3163/1536-5050.97.1.003.
62. Cifuentes M, Davis M, Fernald D, Gunn R, Dickinson P, Cohen DJ. Electronic Health Record Challenges, Workarounds, and Solutions Observed in Practices Integrating Behavioral Health and Primary Care. Journal Of The American Board Of Family Medicine: JABFM. 2015;28 Suppl 1:S63-S72. PMID: 26359473. doi: 10.3122/jabfm.2015.S1.150133.
63. Cunningham A, Johnson F. Exploring trust in online health information: a study of user experiences of patients.co.uk. Health Information And Libraries Journal. 2016;33(4):323-8. PMID: 27870321. doi: 10.1111/hir.12163.
64. Darcy S, Green J, Maxwell H. I've got a mobile phone too! Hard and soft assistive technology customization and supportive call centres for people with disability. Disability And Rehabilitation Assistive Technology. 2017;12(4):341-51. PMID: 27293107. doi: 10.3109/17483107.2016.1167260.
65. Grande D, Mitra N, Shah A, Wan F, Asch DA. Public Preferences About Secondary Uses of Electronic Health Information. Jama Internal Medicine. 2013 Oct;173(19):1798-806. doi: 10.1001/jamainternmed.2013.9166.
66. Gualtieri L, Rosenbluth S, Phillips J. Can a Free Wearable Activity Tracker Change Behavior? The Impact of Trackers on Adults in a Physician-Led Wellness Group. JMIR Research Protocols. 2016;5(4):e237-e. PMID: 27903490.
67. Guo XT, Zhang XF, Sun YQ. The privacy-personalization paradox in mHealth services acceptance of different age groups. Electron Commer Res Appl. 2016 Mar-Apr;16:55-65. doi: 10.1016/j.elerap.2015.11.001.
68. King G, Maxwell J, Karmali A, Hagens S, Pinto M, Williams L, et al. Connecting Families to Their Health Record and Care Team: The Use, Utility, and Impact of a Client/Family Health Portal at a Children's Rehabilitation Hospital. Journal of Medical Internet Research. 2017 Apr;19(4):14. PMID: 28385680. doi: 10.2196/jmir.6811.
69. Kvilén Eriksson E, Sandelius S, Wahlberg AC. Telephone advice nursing: parents' experiences of monitoring calls in children with gastroenteritis. Scandinavian Journal of Caring Sciences. 2015;29(2):333-9. doi: 10.1111/scs.12167.
70. Ottenberg AL, Swetz KM, Mueller LA, Gerhardson S, Mueller PS. "We as Human Beings Get Farther and Farther Apart": the experiences of patients with remote monitoring systems. Heart & Lung: The Journal Of Critical Care. 2013;42(5):313-9. PMID: 23582212. doi: 10.1016/j.hrtlng.2013.03.002.
71. Palazuelos D, Diallo AB, Palazuelos L, Carlile N, Payne JD, Franke MF. User Perceptions of an mHealth Medicine Dosing Tool for Community Health Workers. JMIR Mhealth And Uhealth. 2013;1(1):e2-e. PMID: 25100670. doi: 10.2196/mhealth.2459.
72. Peng W, Kanthawala S, Yuan S, Hussain SA. A qualitative study of user perceptions of mobile health apps. BMC Public Health. 2016;16(1):1158-. PMID: 27842533.
73. Randeree E. Exploring physician adoption of EMRs: A multi-case analysis. Journal of Medical Systems. 2007 Dec;31(6):489-96. doi: 10.1007/s10916-007-9089-5.

74. Robertson A, Cresswell K, Takian A, Petrakaki D, Crowe S, Cornford T, et al. Implementation and adoption of nationwide electronic health records in secondary care in England: qualitative analysis of interim results from a prospective national evaluation. BMJ (Clinical Research Ed). 2010;341:c4564-c. PMID: 20813822. doi: 10.1136/bmj.c4564.
75. Sigler BE. Investigating the Perceptions of Care Coordinators on Using Behavior Theory-Based Mobile Health Technology With Medicaid Populations: A Grounded Theory Study. JMIR Mhealth And Uhealth. 2017;5(3):e36-e. PMID: 28325711. doi: 10.2196/mhealth.5892.
76. van Heerden A, Harris DM, van Rooyen H, Barnabas RV, Ramanathan N, Ngcobo N, et al. Perceived mHealth barriers and benefits for home-based HIV testing and counseling and other care: Qualitative findings from health officials, community health workers, and persons living with HIV in South Africa. Social Science & Medicine (1982). 2017;183:97-105. PMID: 28475904. doi: 10.1016/j.socscimed.2017.04.046.
77. Williamson S, Chalmers K, Beaver K. Patient experiences of nurse-led telephone follow-up following treatment for colorectal cancer. European Journal Of Oncology Nursing: The Official Journal Of European Oncology Nursing Society. 2015;19(3):237-43. PMID: 25529937. doi: 10.1016/j.ejon.2014.11.006.
78. Zhao Y, Heida T, van Wegen EEH, Bloem BR, van Wezel RJA. E-health Support in People with Parkinson's Disease with Smart Glasses: A Survey of User Requirements and Expectations in the Netherlands. Journal Of Parkinson's Disease. 2015;5(2):369-78. PMID: 25855044. doi: 10.3233/JPD-150568.
79. Adler G, Pritchett LR, Kauth MR, Nadorff D. A pilot project to improve access to telepsychotherapy at rural clinics. Telemedicine Journal And E-Health: The Official Journal Of The American Telemedicine Association. 2014;20(1):83-5. PMID: 24168721. doi: 10.1089/tmj.2013.0085.
80. Agbakoba R, McGee-Lennon M, Bouamrane MM, Watson N, Mair F. Implementing a National Scottish Digital Health & Wellbeing Service at Scale: A Qualitative Study of Stakeholders' Views. Studies in health technology and informatics. 2015;216:487-91. PMID: 26262098.
81. Ahern DK, Kreslake JM, Phalen JM. What is eHealth (6): perspectives on the evolution of eHealth research. Journal Of Medical Internet Research. 2006;8(1):e4-e. PMID: 16585029.
82. Asan O, Carayon P, Beasley JW, Montague E. Work system factors influencing physicians' screen sharing behaviors in primary care encounters. International Journal Of Medical Informatics. 2015;84(10):791-8. PMID: 26049312. doi: 10.1016/j.ijmedinf.2015.05.006.
83. Ash JS, Gorman PN, Lavelle M, Stavri PZ, Lyman J, Fournier L, et al. Perceptions of Physician Order Entry: Results of a Cross-Site Qualitative Study. Methods of Information in Medicine. 2003;42(4):313-23. PMID: 14534628.
84. Bacchus LJ, Bullock L, Sharps P, Burnett C, Schminkey DL, Buller AM, et al. Infusing Technology Into Perinatal Home Visitation in the United States for Women Experiencing Intimate Partner Violence: Exploring the Interpretive Flexibility of an mHealth Intervention. Journal Of Medical Internet Research. 2016;18(11):e302-e. PMID: 27856405.
85. Bardach SH, Real K, Bardach DR. Perspectives of healthcare practitioners: An exploration of interprofessional communication using electronic medical records. Journal Of Interprofessional Care. 2017;31(3):300-6. PMID: 28151026. doi: 10.1080/13561820.2016.1269312.

86. Bennett-Levy J, Singer J, DuBois S, Hyde K. Translating E-Mental Health Into Practice: What Are the Barriers and Enablers to E-Mental Health Implementation by Aboriginal and Torres Strait Islander Health Professionals? Journal Of Medical Internet Research. 2017;19(1):e1-e. PMID: 28077347. doi: 10.2196/jmir.6269.
87. Boyle L, Grainger R, Hall RM, Krebs JD. Use of and Beliefs About Mobile Phone Apps for Diabetes Self-Management: Surveys of People in a Hospital Diabetes Clinic and Diabetes Health Professionals in New Zealand. JMIR Mhealth And Uhealth. 2017;5(6):e85-e. PMID: 28666975. doi: 10.2196/mhealth.7263.
88. Bradford NK, Young J, Armfield NR, Herbert A, Smith AC. Home telehealth and paediatric palliative care: clinician perceptions of what is stopping us? BMC Palliative Care. 2014;13:29-. PMID: 24963287. doi: 10.1186/1472-684X-13-29.
89. Braun R, Lasway C, Agarwal S, L'Engle K, Layer E, Silas L, et al. An evaluation of a family planning mobile job aid for community health workers in Tanzania. Contraception. 2016;94(1):27-33. PMID: 27039033. doi: 10.1016/j.contraception.2016.03.016.
90. Bryson M, Tidy N, Smith M, Levy S. An online survey of nurses' perceptions, knowledge and expectations of the National Health Service modernization programme. Journal Of Telemedicine And Telecare. 2005;11 Suppl 1:64-6. PMID: 16035999.
91. Byrne CM, Mercincavage LM, Bouhaddou O, Bennett JR, Pan EC, Botts NE, et al. The Department of Veterans Affairs' (VA) implementation of the Virtual Lifetime Electronic Record (VLER): findings and lessons learned from Health Information Exchange at 12 sites. International Journal Of Medical Informatics. 2014;83(8):537-47. PMID: 24845146. doi: 10.1016/j.ijmedinf.2014.04.005.
92. Clarke A, Adamson J, Sheard L, Cairns P, Watt I, Wright J. Implementing electronic patient record systems (EPRs) into England's acute, mental health and community care trusts: a mixed methods study. BMC Medical Informatics And Decision Making. 2015;15:85-. PMID: 26466894. doi: 10.1186/s12911-015-0204-0.
93. Cook EJ, Randhawa G, Large S, Guppy A, Chater AM, Ali N. Barriers and facilitators to using NHS Direct: a qualitative study of 'users' and 'non-users'. BMC Health Services Research. 2014;14:487-. PMID: 25344061. doi: 10.1186/s12913-014-0487-3.
94. de Witt L. Many older people felt that electronic care surveillance increased their safety and enabled them to live alone in their own homes. Evidence Based Nursing. 2009;12(1):32-. PMID: 19103850. doi: 10.1136/ebn.12.1.32.
95. Dimitropoulos L, Patel V, Scheffler SA, Posnack S. Public attitudes toward health information exchange: perceived benefits and concerns. The American Journal Of Managed Care. 2011;17(12 Spec No.):SP111-SP6. PMID: 22216769.
96. Ditchburn J-L, Marshall A. Renal telemedicine through video-as-a-service delivered to patients on home dialysis: A qualitative study on the renal care team members' experience. Journal Of Renal Care. 2017. PMID: 28614637. doi: 10.1111/jorc.12207.
97. Domingo M, Lupón J, González B, Crespo E, López R, Ramos A, et al. Evaluation of a telemedicine system for heart failure patients: feasibility, acceptance rate, satisfaction and changes in patient behavior: results from the CARME (CAtalan Remote Management Evaluation) study. European journal of cardiovascular nursing : journal of the Working Group on Cardiovascular Nursing of the European Society of Cardiology. 2012;11(4):410-8. PMID: 21402493.

98. Doyle C, Jackson D, Loi S, Malta S, Moore K. Videoconferencing and telementoring about dementia care: Evaluation of a pilot model for sharing scarce old age psychiatry

resources. International Psychogeriatrics. 2016;28(9):1567-74. doi: 10.1017/S1041610216000740.
99. Feufel MA, Robinson FE, Shalin VL. The impact of medical record technologies on collaboration in emergency medicine. International Journal Of Medical Informatics. 2011;80(8):e85-e95. PMID: 21036659. doi: 10.1016/j.ijmedinf.2010.09.008.
100. Finegold P, Mathieson K, Holmes L, Boon M, Cottle M, Donnai D, et al. Is the UK public ready for genetic medicine? Personalized Medicine. 2008;5(1):65-76. PMID: 29783399. doi: 10.2217/17410541.5.1.65.
101. Fisher B, Bhavnani V, Winfield M. How patients use access to their full health records: a qualitative study of patients in general practice. Journal Of The Royal Society Of Medicine. 2009;102(12):539-44. PMID: 19966130. doi: 10.1258/jrsm.2009.090328.
102. Foster J, Jessopp L, Dale J. Concerns and confidence of general practitioners in providing telephone consultations. The British Journal Of General Practice: The Journal Of The Royal College Of General Practitioners. 1999;49(439):111-3. PMID: 10326262.
103. Gadd CS, Ho Y-X, Cala CM, Blakemore D, Chen Q, Frisse ME, et al. User perspectives on the usability of a regional health information exchange. Journal Of The American Medical Informatics Association: JAMIA. 2011;18(5):711-6. PMID: 21622933. doi: 10.1136/amiajnl-2011-000281.
104. Gaylin DS, Moiduddin A, Mohamoud S, Lundeen K, Kelly JA. Public attitudes about health information technology, and its relationship to health care quality, costs, and privacy. Health Services Research. 2011;46(3):920-38. PMID: 21275986. doi: 10.1111/j.1475-6773.2010.01233.x.
105. George S, Hamilton A, Baker RS. How Do Low-Income Urban African Americans and Latinos Feel about Telemedicine? A Diffusion of Innovation Analysis. International Journal Of Telemedicine And Applications. 2012;2012:715194-. PMID: 22997511.
106. Glinkowski W, Pawłowska K, Kozłowska L. Telehealth and telenursing perception and knowledge among university students of nursing in poland. Telemedicine Journal And E-Health: The Official Journal Of The American Telemedicine Association. 2013;19(7):523-9. PMID: 23650941. doi: 10.1089/tmj.2012.0217.
107. Goodwin S, McGuirk M, Reeve C. The impact of video telehealth consultations on professional development and patient care. Australian Journal of Rural Health. 2017;25(3):185-6. PMID: 27076386. doi: 10.1111/ajr.12297.
108. Grande D, Mitra N, Shah A, Wan F, Asch DA. The Importance of Purpose: Moving Beyond Consent in the Societal Use of Personal Health Information. Ann Intern Med. 2014 Dec;161(12):855-U37. PMID: 25506854.
109. Grunloh C, Cajander A, Myreteg G. "The Record is Our Work Tool!"-Physicians' Framing of a Patient Portal in Sweden. Journal of Medical Internet Research. 2016 Jun;18(6):14. PMID: 27349531. doi: 10.2196/jmir.5705.
110. Jung M-L, Loria K. Acceptance of Swedish e-health services. Journal Of Multidisciplinary Healthcare. 2010;3:55-63. PMID: 21289860. doi: 10.2147/JMDH.S9159.
111. Kayyali R, Hesso I, Mahdi A, Hamzat O, Adu A, Nabhani Gebara S. Telehealth: misconceptions and experiences of healthcare professionals in England. The International Journal Of Pharmacy Practice. 2017;25(3):203-9. PMID: 28261891. doi: 10.1111/ijpp.12340.
112. Khatun F, Heywood AE, Ray PK, Bhuiya A, Liaw S-T. Community readiness for adopting mHealth in rural Bangladesh: A qualitative exploration. International Journal Of Medical Informatics. 2016;93:49-56. PMID: 27435947. doi: 10.1016/j.ijmedinf.2016.05.010.

113. Mills J, Woods C, Hitchins M, Summers G. Specialist nurses' experiences of using ‘The Viewer', a consolidated electronic medical records system: a pre-post implementation survey. Australian Journal of Advanced Nursing. 2015;33(1):6-13.
114. Mistry H, Sauer J. Psychiatrists and electronic patient records: The South London and Maudsley experience. Psychiatric Bulletin. 2009;33(9):325-8. doi: 10.1192/pb.bp.108.019588.
115. Miyamoto K, Iwakuma M, Nakayama T. Experiences and attitudes of residents regarding a community-based genome cohort study in Japan: a population-based, cross-sectional study. Bmc Medical Genomics. 2016 Mar;9:10. PMID: 28081699. doi: 10.1186/s12920-016-0175-8.
116. Nhavoto JA, Gronlund A, Klein GO. Mobile health treatment support intervention for HIV and tuberculosis in Mozambique: Perspectives of patients and healthcare workers. Plos One. 2017 Apr;12(4):13. PMID: 28419149. doi: 10.1371/journal.pone.0176051.
117. Nilsson C, Skär L, Söderberg S. Swedish District Nurses' experiences on the use of information and communication technology for supporting people with serious chronic illness living at home--a case study. Scandinavian Journal Of Caring Sciences. 2010;24(2):259-65. PMID: 20030770. doi: 10.1111/j.1471-6712.2009.00715.x.
118. Oliveri S, Masiero M, Arnaboldi P, Cutica I, Fioretti C, Pravettoni G. Health Orientation, Knowledge, and Attitudes toward Genetic Testing and Personalized Genomic Services: Preliminary Data from an Italian Sample. Biomed Research International. 2016;2016:6824581-. PMID: 28105428. doi: 10.1155/2016/6824581.
119. Powell KP, Christianson CA, Cogswell WA, Dave G, Verma A, Eubanks S, et al. Educational Needs of Primary Care Physicians Regarding Direct-to-Consumer Genetic Testing. Journal of Genetic Counseling. 2012 Jun;21(3):469-78. PMID: 22207397. doi: 10.1007/s10897-011-9471-9.
120. Power JMH, Spina SP, Forbes DA, Harder CK, Lalli SL, Loewen PS, et al. Integration of Smartphones into clinical pharmacy practice: An evaluation of the impact on pharmacists' efficiency. Health Policy and Technology. 2014;3(4):296-305. doi: 10.1016/j.hlpt.2014.08.002.
121. Rasmussen BSB, Jensen LK, Froekjaer J, Kidholm K, Kensing F, Yderstraede KB. A qualitative study of the key factors in implementing telemedical monitoring of diabetic foot ulcer patients. International Journal Of Medical Informatics. 2015;84(10):799-807. PMID: 26093794. doi: 10.1016/j.ijmedinf.2015.05.012.
122. Rathert C, Porter TH, Mittler JN, Fleig-Palmer M. Seven years after Meaningful Use: Physicians' and nurses' experiences with electronic health records. Health Care Management Review. 2017. PMID: 28614166. doi: 10.1097/HMR.0000000000000168.
123. Ray KN, Ashcraft LE, Mehrotra A, Miller E, Kahn JM. Family Perspectives on Telemedicine for Pediatric Subspecialty Care. Telemedicine Journal And E-Health: The Official Journal Of The American Telemedicine Association. 2017. PMID: 28430021. doi: 10.1089/tmj.2016.0236.
124. Tews M, Brennan K, Begaz T, Treat R. Medical student case presentation performance and perception when using mobile learning technology in the emergency department. Medical Education Online. 2011;16. PMID: 22013378. doi: 10.3402/meo.v16i0.7327.
125. Thondoo M, Strachan DL, Nakirunda M, Ndima S, Muiambo A, Källander K, et al. Potential Roles of Mhealth for Community Health Workers: Formative Research With End Users in Uganda and Mozambique. JMIR Mhealth And Uhealth. 2015;3(3):e76-e. PMID: 26206419. doi: 10.2196/mhealth.4208.

126. Trondsen MV. Managing Everyday Life: A Qualitative Study of Patients' Experiences of a Web-Based Ulcer Record for Home-Based Treatment. Healthcare (Basel, Switzerland). 2014;2(4):492-504. PMID: 27429289. doi: 10.3390/healthcare2040492.
127. Wagner L, Paquin R, Persky S. Genetics blogs as a public health tool: assessing credibility and influence. Public Health Genomics. 2012;15(3-4):218-25. PMID: 22488465. doi: 10.1159/000336537.
128. Ward L, Innes M. Electronic medical summaries in general practice - Considering the patient's contribution. British Journal of General Practice. 2003;53(489):293-7.
129. Wibe T, Slaughter L. Patients reading their health records - what emotional factors are involved? Studies in health technology and informatics. 2009;146:174-8. PMID: 19592830.
130. Wolff JL, Darer JD, Berger A, Clarke D, Green JA, Stametz RA, et al. Inviting patients and care partners to read doctors' notes: OpenNotes and shared access to electronic medical records. Journal of the American Medical Informatics Association. 2017 Apr;24(E1):E166-E72. PMID: 27497795. doi: 10.1093/jamia/ocw108.
131. Wozney L, Newton AS, Gehring ND, Bennett K, Huguet A, Hartling L, et al. Implementation of eMental Health care: viewpoints from key informants from organizations and agencies with eHealth mandates. BMC Medical Informatics And Decision Making. 2017;17(1):78-. PMID: 28577543. PMID: 28577543. doi: 10.1186/s12911-017-0474-9.
132. Albert NM, Dinesen B, Spindler H, Southard J, Bena JF, Catz S, et al. Factors associated with telemonitoring use among patients with chronic heart failure. Journal Of Telemedicine And Telecare. 2017;23(2):283-91. PMID: 26869144. doi: 10.1177/1357633X16630444.
133. Ancker JS, Brenner S, Richardson JE, Silver M, Kaushal R. Trends in public perceptions of electronic health records during early years of meaningful use. The American Journal Of Managed Care. 2015;21(8):e487-e93. PMID: 26625503.
134. Andrews L, Gajanayake R, Sahama T. The Australian general public's perceptions of having a personally controlled electronic health record (PCEHR). International Journal Of Medical Informatics. 2014;83(12):889-900. PMID: 25200198. doi: 10.1016/j.ijmedinf.2014.08.002.
135. Becevic M, Boren S, Mutrux R, Shah Z, Banerjee S. User Satisfaction With Telehealth: Study of Patients, Providers, and Coordinators. The Health Care Manager. 2015;34(4):337-49. PMID: 26506296. doi: 10.1097/HCM.0000000000000081.
136. Botts NE, Horan TA, Thoms BP. HealthATM: personal health cyberinfrastructure for underserved populations. American Journal Of Preventive Medicine. 2011;40(5 Suppl 2):S115-S22. PMID: 21521584. doi: 10.1016/j.amepre.2011.01.016.
137. Bull TP, Dewar AR, Malvey DM, Szalma JL. Considerations for the Telehealth Systems of Tomorrow: An Analysis of Student Perceptions of Telehealth Technologies. JMIR Medical Education. 2016;2(2):e11-e. PMID: 27731865.
138. Deng ZH. Understanding public users' adoption of mobile health service. Int J Mob Commun. 2013;11(4):351-73. doi: 10.1504/ijmc.2013.055748.
139. Dick JJ, Nundy S, Solomon MC, Bishop KN, Chin MH, Peek ME. Feasibility and usability of a text message-based program for diabetes self-management in an urban African-American population. Journal Of Diabetes Science And Technology. 2011;5(5):1246-54. PMID: 22027326.
140. Flynn D, Gregory P, Makki H, Gabbay M. Expectations and experiences of eHealth in primary care: A qualitative practice-based investigation. International Journal of Medical Informatics. 2009 Sep;78(9):588-604. doi: 10.1016/j.ijmedinf.2009.03.008.

141. Gibson F, Miller M, Kearney N. Technology into practice: young people's, parents' and nurses' perceptions of WISECARE+. Paediatric Nursing. 2007;19(10):31-4. PMID: 18196857.
142. Honeyman A, Cox B, Fisher B. Potential impacts of patient access to their electronic care records. Informatics in Primary Care. 2005;13(1):55-60. PMID: 15949176.
143. Hsieh P-J. Physicians' acceptance of electronic medical records exchange: an extension of the decomposed TPB model with institutional trust and perceived risk. International Journal Of Medical Informatics. 2015;84(1):1-14. PMID: 25242228. doi: 10.1016/j.ijmedinf.2014.08.008.
144. Jennings A, Powell J, Armstrong N, Sturt J, Dale J. A virtual clinic for diabetes self-management: pilot study. Journal Of Medical Internet Research. 2009;11(1):e10-e. PMID: 21821504. doi: 10.2196/jmir.1111.
145. Kim NE, Han SS, Yoo KH, Yun EK. The impact of user's perceived ability on online health information acceptance. Telemedicine Journal And E-Health: The Official Journal Of The American Telemedicine Association. 2012;18(9):703-8. PMID: 23072632. doi: 10.1089/tmj.2011.0277.
146. Lanseng EJ, Andreassen TW. Electronic healthcare: a study of people's readiness and attitude toward performing self-diagnosis. Int J Serv Ind Manage. 2007;18(3-4):394-417. doi: 10.1108/09564230710778155.
147. Levy ME, Watson CC, Wilton L, Criss V, Kuo I, Glick SN, et al. Acceptability of a Mobile Smartphone Application Intervention to Improve Access to HIV Prevention and Care Services for Black Men Who Have Sex with Men in the District of Columbia. Digital Culture & Education. 2015;7(2):169-91. PMID: 26594251.
148. Luger TM, Houston TK, Suls J. Older adult experience of online diagnosis: results from a scenario-based think-aloud protocol. Journal Of Medical Internet Research. 2014;16(1):e16-e. PMID: 24434479. doi: 10.2196/jmir.2924.
149. Mou J, Shin D-H, Cohen J. Understanding trust and perceived usefulness in the consumer acceptance of an e-service: A longitudinal investigation. Behaviour & Information Technology. 2017;36(2):125-39. doi: 10.1080/0144929X.2016.1203024.
150. Nitsch M, Dimopoulos CN, Flaschberger E, Saffran K, Kruger JF, Garlock L, et al. A Guided Online and Mobile Self-Help Program for Individuals With Eating Disorders: An Iterative Engagement and Usability Study. Journal Of Medical Internet Research. 2016;18(1):e7-e. PMID: 26753539. doi: 10.2196/jmir.4972.
151. Pathipati AS, Ko JM. Implementation and evaluation of Stanford Health Care direct-care teledermatology program. SAGE Open Medicine. 2016;4. doi: 10.1177/2050312116659089.
152. Pecina JL, Vickers KS, Finnie DM, Hathaway JC, Hanson GJ, Takahashi PY. Telemonitoring increases patient awareness of health and prompts health-related action: initial evaluation of the TELE-ERA study. Telemedicine Journal And E-Health: The Official Journal Of The American Telemedicine Association. 2011;17(6):461-6. PMID: 21612521. doi: 10.1089/tmj.2010.0213.
153. Rowsell A, Muller I, Murray E, Little P, Byrne CD, Ganahl K, et al. Views of People With High and Low Levels of Health Literacy About a Digital Intervention to Promote Physical Activity for Diabetes: A Qualitative Study in Five Countries. Journal Of Medical Internet Research. 2015;17(10):e230-e. PMID: 26459743. doi: 10.2196/jmir.4999.
154. Tlach L, Thiel J, Härter M, Liebherz S, Dirmaier J. Acceptance of the German e-mental health portal www.psychenet.de: an online survey. Peerj. 2016;4:e2093-e. PMID: 27547515. doi: 10.7717/peerj.2093.

155. van Velthoven MH, Li Y, Wang W, Chen L, Du X, Wu Q, et al. Prevalence of mobile phones and factors influencing usage by caregivers of young children in daily life and for health care in rural China: a mixed methods study. Plos One. 2015;10(3):e0116216-e. PMID: 25789477. doi: 10.1371/journal.pone.0116216.
156. Wang SH. Web-Based Medical Service: Technology Attractiveness, Medical Creditability, Information Source, and Behavior Intention. Journal of Medical Internet Research. 2017 Aug;19(8):11. PMID: 28768608. doi: 10.2196/jmir.8114.
157. Bhuyan SS, Lu N, Chandak A, Kim H, Wyant D, Bhatt J, et al. Use of Mobile Health Applications for Health-Seeking Behavior Among US Adults. Journal Of Medical Systems. 2016;40(6):153-. PMID: 27147516. doi: 10.1007/s10916-016-0492-7.
158. Carere DA, Kraft P, Kaphingst KA, Roberts JS, Green RC. Consumers report lower confidence in their genetics knowledge following direct-to-consumer personal genomic testing. Genetics In Medicine: Official Journal Of The American College Of Medical Genetics. 2016;18(1):65-72. PMID: 25812042. doi: 10.1038/gim.2015.34.
159. Li J, Liu M, Liu X, Ma L. Why and When do Patients Use e-Consultation Services? The Trust and Resource Supplementary Perspectives. Telemedicine Journal And E-Health: The Official Journal Of The American Telemedicine Association. 2017. PMID: 28686084. doi: 10.1089/tmj.2016.0268.
160. Lupo PJ, Robinson JO, Diamond PM, Jamal L, Danysh HE, Blumenthal-Barby J, et al. Patients' perceived utility of whole-genome sequencing for their healthcare: findings from the MedSeq project. Personalized Medicine. 2016;13(1):13-20. PMID: 27019659.
161. Mano R. Online health information, situational effects and health changes among e-patients in israel: A ‘push/pull’ perspective. Health Expectations: An International Journal of Public Participation in Health Care & Health Policy. 2015;18(6):2489-500. doi: 10.1111/hex.12218.
162. Mead N, Varnam R, Rogers A, Roland M. What predicts patients' interest in the Internet as a health resource in primary care in England? Journal Of Health Services Research & Policy. 2003;8(1):33-9. PMID: 12683432.
163. Mou J, Shin D-H, Cohen J. Health beliefs and the valence framework in health information seeking behaviors. Information Technology & People. 2016;29(4):876-900. doi: 10.1108/ITP-06-2015-0140.
164. Rogers A, Mead N. More than technology and access: primary care patients' views on the use and non-use of health information in the Internet age. Health & Social Care in the Community. 2004;12(2):102-10. PMID: 19777718.
165. Srinivasan D. The impact of trust in E-government on electronic health literacy. World Medical and Health Policy. 2014;6(1):22-38. doi: 10.1002/wmh3.86.
166. Winkelman WJ, Leonard KJ, Rossos PG. Patient-perceived usefulness of online electronic medical records: employing grounded theory in the development of information and communication technologies for use by patients living with chronic illness. Journal Of The American Medical Informatics Association: JAMIA. 2005;12(3):306-14. PMID: 15684128.
167. Zhao J, Ha S, Widdows R. Building trusting relationships in online health communities. Cyberpsychology, Behavior, and Social Networking. 2013;16(9):650-7. doi: 10.1089/cyber.2012.0348.
168. Peña-Purcell N. Hispanics' use of Internet health information: an exploratory study. Journal of the Medical Library Association. 2008;96(2):101-7. PMID: 18379664.
169. Adkins EC, Zalta AK, Boley RA, Glover A, Karnik NS, Schueller SM. Exploring the potential of technology-based mental health services for homeless youth: A qualitative

study. Psychological Services. 2017;14(2):238-45. PMID: 28481610. doi: 10.1037/ser0000120.
170. Aitken M, Cunningham-Burley S, Pagliari C. Moving from trust to trustworthiness: Experiences of public engagement in the Scottish Health Informatics Programme. Science & Public Policy. 2016;43(5):713-23. PMID: 28066123. doi: 10.1093/scipol/scv075.
171. Atienza AA, Zarcadoolas C, Vaughon W, Hughes P, Patel V, Chou W-YS, et al. Consumer Attitudes and Perceptions on mHealth Privacy and Security: Findings From a Mixed-Methods Study. Journal Of Health Communication. 2015;20(6):673-9. PMID: 25868685. doi: 10.1080/10810730.2015.1018560.
172. Brown SD, Grijalva CS, Ferrara A. Leveraging EHRs for patient engagement: Perspectives on tailored program outreach. American Journal of Managed Care. 2017;23(7):e223-e30.
173. Papoutsi C, Reed JE, Marston C, Lewis R, Majeed A, Bell D. Patient and public views about the security and privacy of Electronic Health Records (EHRs) in the UK: results from a mixed methods study. BMC Medical Informatics And Decision Making. 2015;15:86-. PMID: 26466787. doi: 10.1186/s12911-015-0202-2. PMID: 28850790.
174. Patil S, Lu H, Saunders CL, Potoglou D, Robinson N. Public preferences for electronic health data storage, access, and sharing - evidence from a pan-European survey. Journal of the American Medical Informatics Association. 2016 Nov;23(6):1096-106. doi: 10.1093/jamia/ocw012.
175. Samadbeik M, Gorzin Z, Khoshkam M, Roudbari M. Managing the security of nursing data in the electronic health record. Acta Informatica Medica: AIM: Journal Of The Society For Medical Informatics Of Bosnia & Herzegovina: Casopis Drustva Za Medicinsku Informatiku Bih. 2015;23(1):39-43. PMID: 25870490. doi: 10.5455/aim.2015.23.39-43.
176. Tissera SR, Silva SN. Attitude Towards Health Information Privacy and Electronic Health Records Among Urban Sri Lankan Adults. Studies In Health Technology And Informatics. 2016;225:1003-4. PMID: 27332453.
177. Tjora A, Tran T, Faxvaag A. Privacy vs usability: a qualitative exploration of patients' experiences with secure Internet communication with their general practitioner. Journal Of Medical Internet Research. 2005;7(2):e15-e. PMID: 15998606.
178. Bao Y, Hoque R, Wang S. Investigating the determinants of Chinese adult children's intention to use online health information for their aged parents. International Journal Of Medical Informatics. 2017;102:12-20. PMID: 28495340. doi: 10.1016/j.ijmedinf.2017.01.003.
179. Bevan JL, Lynch JA, Dubriwny TN, Harris TM, Achter PJ, Reeder AL, et al. Informed lay preferences for delivery of racially varied pharmacogenomics. Genetics in Medicine. 2003 Sep-Oct;5(5):393-9. doi: 10.1097/01.gim.0000087989.12317.3f.
180. Critchley C, Nicol D, Otlowski M, Chalmers D. Public reaction to direct-to-consumer online genetic tests: Comparing attitudes, trust and intentions across commercial and conventional providers. Public Underst Sci. 2015 Aug;24(6):731-50. PMID: 24553439. doi: 10.1177/0963662513519937.
181. Greenhalgh T, Wood GW, Bratan T, Stramer K, Hinder S. Patients' attitudes to the summary care record and HealthSpace: Qualitative study. BMJ. 2008;336(7656):1290-5. doi: 10.1136/bmj.a114.
182. Huizenga CR, Lowstuter K, Banks KC, Lagos VI, Vandergon VO, Weitzel JN. Evolving perspectives on genetic discrimination in health insurance among health care providers. Familial Cancer. 2010;9(2):253-60. PMID: 19967457. doi: 10.1007/s10689-009-9308-y.

183. Maiorana A, Steward WT, Koester KA, Pearson C, Shade SB, Chakravarty D, et al. Trust, confidentiality, and the acceptability of sharing HIV-related patient data: lessons learned from a mixed methods study about Health Information Exchanges. Implementation Science: IS. 2012;7:34-. PMID: 22515736. doi: 10.1186/1748-5908-7-34.
184. Neville RG, Greene AC, Lewis S. Patient and health care professional views and experiences of computer agent-supported health care. Informatics in Primary Care. 2006;14(1):11-5. PMID: 16848962.
185. Weitzman ER, Kelemen S, Kaci L, Mandl KD. Willingness to share personal health record data for care improvement and public health: a survey of experienced personal health record users. BMC Medical Informatics And Decision Making. 2012;12:39-. PMID: 22616619. doi: 10.1186/1472-6947-12-39.
186. Bullock A, Dimond R, Webb K, Lovatt J, Hardyman W, Stacey M. How a mobile app supports the learning and practice of newly qualified doctors in the UK: an intervention study. BMC Medical Education. 2015;15:71-. PMID: 25889996. doi: 10.1186/s12909-015-0356-8.
187. Carroll JC, Makuwaza T, Manca DP, Sopcak N, Permaul JA, O'Brien MA, et al. Primary care providers' experiences with and perceptions of personalized genomic medicine. Canadian Family Physician Medecin De Famille Canadien. 2016;62(10):e626-e35. PMID: 27737998.
188. Lee K, Hoti K, Hughes JD, Emmerton LM. Consumer Use of "Dr Google": A Survey on Health Information-Seeking Behaviors and Navigational Needs. Journal Of Medical Internet Research. 2015;17(12):e288-e. PMID: 26715363. doi: 10.2196/jmir.4345.
189. Legido-Quigley H, Doering N, McKee M. Challenges facing teleradiology services across borders in the European union: A qualitative study. Health Policy and Technology. 2014;3(3):160-6. doi: 10.1016/j.hlpt.2014.04.001.
190. Beaver K, Williamson S, Chalmers K. Telephone follow-up after treatment for breast cancer: views and experiences of patients and specialist breast care nurses. Journal of Clinical Nursing. 2010;19(19/20):2916-24. PMID: 20649914. doi: 10.1111/j.1365-2702.2010.03197.x.
191. Fletcher-Tomenius LJ, Vossler A. Trust in online therapeutic relationships: The therapist’s experience. Counselling Psychology Review. 2009;24(2):24-33.
192. Gauld R, Williams S. Use of the Internet for health information: a study of Australians and New Zealanders. Informatics For Health & Social Care. 2009;34(3):149-58. PMID: 19670005. doi: 10.1080/17538150903102448.
193. Holmström IK, Nokkoudenmäki M-B, Zukancic S, Sundler AJ. It is important that they care - older persons' experiences of telephone advice nursing. Journal Of Clinical Nursing. 2016;25(11-12):1644-53. PMID: 26961337. doi: 10.1111/jocn.13173.
194. Huh J, DeLorme DE, Reid LN. Factors Affecting Trust in On-line Prescription Drug Information and Impact of Trust on Behavior Following Exposure to DTC Advertising. Journal of Health Communication. 2005;10(8):711-31. doi: 10.1080/10810730500326716.
195. Huygens MWJ, Vermeulen J, Friele RD, van Schayck OCP, de Jong JD, de Witte LP. Internet Services for Communicating With the General Practice: Barely Noticed and Used by Patients. Interactive Journal of Medical Research. 2015 Oct-Dec;4(4):22-33. PMID: 26601596. doi: 10.2196/ijmr.4245.
196. Kim H, Paige Powell M, Bhuyan SS, Bhuyan SS. Seeking Medical Information Using Mobile Apps and the Internet: Are Family Caregivers Different from the General Public?

Journal Of Medical Systems. 2017;41(3):38-. PMID: 28101781. doi: 10.1007/s10916-017-0684-9.
197. Krebs P, Duncan DT. Health App Use Among US Mobile Phone Owners: A National Survey. JMIR Mhealth And Uhealth. 2015;3(4):e101-e. PMID: 26537656. doi: 10.2196/mhealth.4924.
198. Lipp A, Davis R, Peter R, Davies J. The use of social media among health care professionals within an online postgraduate diabetes diploma course. Practical Diabetes. 2014;31(1):14-7a. doi: 10.1002/pdi.1821.
199. Anthony DL, Campos-Castillo C. A looming digital divide? Group differences in the perceived importance of electronic health records. Info Commun Soc. 2015 Jul;18(7):832-46. doi: 10.1080/1369118x.2015.1006657.
200. Fitton C, Fitton R, Hannan A, Fisher B, Morgan L, Halsall D. The impact of patient record access on appointments and telephone calls in two english general practices: A populationbased study. London Journal of Primary Care. 2014 (6):8-15. PMID: 25949705.
201. Fogel AL, Teng JMC. Pediatric teledermatology: a survey of usage, perspectives, and practice. Pediatric Dermatology. 2015;32(3):363-8. PMID: 25691131. doi: 10.1111/pde.12533.
202. Logue MD, Effken JA. An exploratory study of the personal health records adoption model in the older adult with chronic illness. Informatics in Primary Care. 2012;20(3):151-69. PMID: 23710840.
203. Mahlmann L, Rocke C, Brand A, Hafen E, Vayena E. Attitudes towards personal genomics among older Swiss adults: An exploratory study. Appl Transl Genomics. 2016 Mar;8:9-15. PMID: 27047754. doi: 10.1016/j.atg.2016.01.009.
204. McNally G, Frey R, Crossan M. Nurse manager and student nurse perceptions of the use of personal smartphones or tablets and the adjunct applications, as an educational tool in clinical settings. Nurse Education In Practice. 2017;23:1-7. PMID: 28137514. doi: 10.1016/j.nepr.2016.12.004.
205. Newman L, Biedrzycki K, Baum F. Digital technology use among disadvantaged Australians: implications for equitable consumer participation in digitally-mediated communication and information exchange with health services. Australian Health Review: A Publication Of The Australian Hospital Association. 2012;36(2):125-9. PMID: 22624630. doi: 10.1071/AH11042.
206. Bradford NK, Caffery LJ, Smith AC. Awareness, experiences and perceptions of telehealth in a rural Queensland community. BMC Health Services Research. 2015;15:427-. PMID: 26416464. doi: 10.1186/s12913-015-1094-7.
207. Cho J, Lee HE, Quinlan M. Complementary relationships between traditional media and health apps among american college students. Journal Of American College Health: J Of ACH. 2015;63(4):248-57. PMID: 25692247. doi: 10.1080/07448481.2015.1015025.
208. Dye T, Li DM, Demment M, Groth S, Fernandez D, Dozier A, et al. Sociocultural variation in attitudes toward use of genetic information and participation in genetic research by race in the United States: implications for precision medicine. Journal of the American Medical Informatics Association. 2016 Jul;23(4):782-6. doi: 10.1093/jamia/ocv214.
209. Leibfried M, Pisano M. The utilization of a simulated electronic medical record in an introductory pharmacy practice experience. Currents in Pharmacy Teaching and Learning. 2016;8(4):458-62. doi: 10.1016/j.cptl.2016.03.015.
210. McCarty CA, Nair A, Austin DM, Giampietro PF. Informed consent and subject motivation to participate in a large, population-based genomics study: the Marshfield Clinic

Personalized Medicine Research Project. Community Genetics. 2007;10(1):2-9. PMID: 17167244.
211. Odeh B, Kayyali R, Nabhani-Gebara S, Philip N. Implementing a telehealth service: nurses' perceptions and experiences. British Journal Of Nursing (Mark Allen Publishing). 2014;23(21):1133-7. PMID: 25426527. doi: 10.12968/bjon.2014.23.21.1133.
212. Prochaska MT, Press VG, Meltzer DO, Arora VM. Patient Perceptions of Wearable Face-Mounted Computing Technology and the Effect on the Doctor-Patient Relationship. Applied Clinical Informatics. 2016;7(4):946-53. PMID: 27730249.
213. Sanders C, Rogers A, Bowen R, Bower P, Hirani S, Cartwright M, et al. Exploring barriers to participation and adoption of telehealth and telecare within the Whole System Demonstrator trial: a qualitative study. BMC Health Services Research. 2012;12:220-. PMID: 22834978. doi: 10.1186/1472-6963-12-220.
214. Warriner S, Martinez A. Telemedicine: supporting normality in midwife-led community units. British Journal of Midwifery. 2005;13(10):654-8.
215. Woodward MA, Ple-Plakon P, Blachley T, Musch DC, Newman-Casey PA, De Lott LB, et al. Eye care providers' attitudes towards tele-ophthalmology. Telemedicine Journal And E-Health: The Official Journal Of The American Telemedicine Association. 2015;21(4):271-3. PMID: 25635290. doi: 10.1089/tmj.2014.0115.
216. Cash T, Desbrow B, Leveritt M, Ball L. Utilization and preference of nutrition information sources in Australia. Health Expectations. 2015;18(6):2288-95. PMID: 24798108. doi: 10.1111/hex.12198.
217. Church EA, Heath OJ, Curran VR, Bethune C, Callanan TS, Cornish PA. Rural professionals' perceptions of interprofessional continuing education in mental health. Health & Social Care in the Community. 2010;18(4):433-43. doi: 10.1111/j.1365-2524.2010.00938.x.
218. Cranen K, Drossaert CHC, Brinkman ES, Braakman-Jansen ALM, Ijzerman MJ, Vollenbroek-Hutten MMR. An exploration of chronic pain patients' perceptions of home telerehabilitation services. Health Expectations: An International Journal Of Public Participation In Health Care And Health Policy. 2012;15(4):339-50. PMID: 21348905. doi: 10.1111/j.1369-7625.2011.00668.x.
219. Egea JMO, González MVR. Explaining physicians' acceptance of EHCR systems: An extension of TAM with trust and risk factors. Computers in Human Behavior. 2011;27(1):319-32. doi: 10.1016/j.chb.2010.08.010.
220. Gammon D, Sørlie T, Bergvik S, Høifødt TS. Psychotherapy supervision conducted by videoconferencing: a qualitative study of users' experiences. Journal Of Telemedicine And Telecare. 1998;4 Suppl 1:33-5. PMID: 9640727.
221. Hewitt H, Gafaranga J, McKinstry B. Comparison of face-to-face and telephone consultations in primary care: Qualitative analysis. British Journal of General Practice. 2010;60(574):e201-e12. PMID: 20423575. doi: 10.3399/bjgp10X501831.
222. Holmström I, Höglund AT. The faceless encounter: ethical dilemmas in telephone nursing. Journal Of Clinical Nursing. 2007;16(10):1865-71. PMID: 17880475.
223. Holst A, Nejati S, Björkelund C, Eriksson MCM, Hange D, Kivi M, et al. Patients' experiences of a computerised self-help program for treating depression – a qualitative study of Internet mediated cognitive behavioural therapy in primary care. Scandinavian Journal of Primary Health Care. 2017;35(1):46-53. PMID: 28277055. doi: 10.1080/02813432.2017.1288813.

224. Musiat P, Goldstone P, Tarrier N. Understanding the acceptability of e-mental health--attitudes and expectations towards computerised self-help treatments for mental health problems. BMC Psychiatry. 2014;14:109-. PMID: 24725765. doi: 10.1186/1471-244X-14-109.
225. Pennbridge J, Moya R, Rodrigues L. Questionnaire survey of California consumers' use and rating of sources of health care information including the Internet. The Western Journal Of Medicine. 1999;171(5-6):302-5. PMID: 10639865.
226. Walivaara BM, Andersson S, Axelsson K. Views on Technology Among People in Need of Health Care at Home. International Journal of Circumpolar Health. 2009 Apr;68(2):158-69. doi: 10.3402/ijch.v68i2.18326.
227. Dawson A, Joof BM. Seeing, thinking and acting against malaria -- a new approach to health worker training in rural Gambia. Education for Health: Change in Learning & Practice (Taylor & Francis Ltd). 2005;18(3):387-94. PMID: 16283819.
228. Guise V, Wiig S. Perceptions of telecare training needs in home healthcare services: a focus group study. BMC Health Services Research. 2017;17(1):164-. PMID: 28231852. doi: 10.1186/s12913-017-2098-2.
229. Keddie Z, Jones R. Information communications technology in general practice: cross-sectional survey in London. Informatics in Primary Care. 2005;13(2):113-23. PMID: 15992496.
230. Lærum H, Bremer S, Bergan S, Grünfeld T. A taste of individualized medicine: physicians' reactions to automated genetic interpretations. Journal Of The American Medical Informatics Association: JAMIA. 2014;21(e1):e143-e6. PMID: 24001515. doi: 10.1136/amiajnl-2012-001587.
231. Lee K, Hoti K, Hughes JD, Emmerton L. Dr Google and the consumer: a qualitative study exploring the navigational needs and online health information-seeking behaviors of consumers with chronic health conditions. Journal Of Medical Internet Research. 2014;16(12):e262-e. PMID: 25470306. doi: 10.2196/jmir.3706.
232. Ochieng OG, Hosoi R. Factors influencing diffusion of electronic medical records: a case study in three healthcare institutions in Japan. Health Information Management Journal. 2005;34(4):120-9. PMID: 18216416.
233. van der Vaart R, Drossaert CHC, Taal E, Drossaers-Bakker KW, Vonkeman HE, van de Laar M. Impact of patient-accessible electronic medical records in rheumatology: use, satisfaction and effects on empowerment among patients. Bmc Musculoskeletal Disorders. 2014 Mar;15:9. doi: 10.1186/1471-2474-15-102.
234. Wibe T, Ekstedt M, Hellesø R, Øyri K, Slaughter L. Clinical documentation as a source of information for patients - possibilities and limitations. Studies in health technology and informatics. 2013;192:793-7. PMID:23920666.
235. Hung SY, Tsai JCA, Chuang CC. Investigating primary health care nurses' intention to use information technology: An empirical study in Taiwan. Decision Support Systems. 2014 Jan;57:331-42. doi: 10.1016/j.dss.2013.09.016.
236. Albright K, Krantz MJ, Backlund Jarquín P, DeAlleaume L, Coronel-Mockler S, Estacio RO. Health promotion text messaging preferences and acceptability among the medically underserved. Health Promotion Practice. 2015;16(4):523-32. PMID: 25586133. doi: 10.1177/1524839914566850.
237. Ancker JS, Edwards AM, Miller MC, Kaushal R. Consumer perceptions of electronic health information exchange. American Journal Of Preventive Medicine. 2012;43(1):76-80. PMID: 22704751. doi: 10.1016/j.amepre.2012.02.027.

238. Ancker JS, Witteman HO, Hafeez B, Provencher T, Van de Graaf M, Wei E. "You Get Reminded You're a Sick Person": Personal Data Tracking and Patients With Multiple Chronic Conditions. Journal of Medical Internet Research. 2015 Aug;17(8):12. PMID: 26290186. doi: 10.2196/jmir.4209.
239. Campos-Castillo C, Anthony DL. The double-edged sword of electronic health records: implications for patient disclosure. Journal of the American Medical Informatics Association. 2015 Apr;22(E1):E130-E40. PMID: 25059953. doi: 10.1136/amiajnl-2014-002804.
240. Chang HH, Chang CS. An assessment of technology-based service encounters & network security on the e-health care systems of medical centers in Taiwan. BMC Health Services Research. 2008;8:87-. PMID: 18419820. doi: 10.1186/1472-6963-8-87.
241. Hwang HG, Han HE, Kuo KM, Liu CF. The Differing Privacy Concerns Regarding Exchanging Electronic Medical Records of Internet Users in Taiwan. Journal of Medical Systems. 2012 Dec;36(6):3783-93. PMID: 22527781. doi: 10.1007/s10916-012-9851-1.
242. Karwig G, Chambers D. E-mental health on-campus: College students' views of online help-seeking. Annual Review of CyberTherapy and Telemedicine. 2016;14:58-64. Interactive media Institute. ISBN 1554-8716.
243. Matharu J, Hale B, Ammar M, Brennan PA. Short message service (SMS) texting as a method of communication during on call: prevalence and experience of medical staff in a large acute NHS Trust in the UK. The British Journal Of Oral & Maxillofacial Surgery. 2016;54(8):863-7. PMID: 27400819. doi: 10.1016/j.bjoms.2016.05.034.
244. Patel V, Beckjord E, Moser RP, Hughes P, Hesse BW. The role of health care experience and consumer information efficacy in shaping privacy and security perceptions of medical records: national consumer survey results. JMIR Medical Informatics. 2015;3(2):e14-e. PMID: 25843686. doi: 10.2196/medinform.3238.
245. Usher W. General practitioners' understanding pertaining to reliability, interactive and usability components associated with health websites. Behaviour & Information Technology. 2009;28(1):39-44.
246. Vodicka E, Mejilla R, Leveille SG, Ralston JD, Darer JD, Delbanco T, et al. Online access to doctors' notes: patient concerns about privacy. Journal Of Medical Internet Research. 2013;15(9):e208-e. PMID: 24072335. doi: 10.2196/jmir.2670.
247. Nicholas D, Huntington P, Williams P, Gunter B. Perceptions of the authority of health information. Case study: digital interactive television and the Internet. Health Information And Libraries Journal. 2003;20(4):215-24. PMID: 14641494.
248. Tsai C-H, Kuo Y-M, Uei S-L. Influences of satisfaction with telecare and family trust in older Taiwanese people. International Journal Of Environmental Research And Public Health. 2014;11(2):1359-68. PMID: 24473111. doi: 10.3390/ijerph110201359.
249. Uei S-L, Tsai C-H, Yang M-S. Telecare service use among Taiwanese aged 60 and over: Satisfaction, trust, and continued use intention. Social Behavior and Personality. 2013;41(8):1309-18. doi: 10.2224/sbp.2013.41.8.1309.
250. Greenberg ME, Schultz C. Telephone nursing: client experiences and perceptions. Nursing Economics. 2002;20(4):181-7.
251. McCord G, Pendleton BF, Schrop SL, Weiss L, Stockton L, Hamrich LM. Assessing the impact on patient-physician interaction when physicians use personal digital assistants: a Northeastern Ohio Network (NEON) study. Journal Of The American Board Of Family Medicine: JABFM. 2009;22(4):353-9. PMID: 19587248. doi: 10.3122/jabfm.2009.04.080056.

252. Bates BR, Romina S, Ahmed R, Hopson D. The effect of source credibility on consumers' perceptions of the quality of health information on the Internet. Medical Informatics and the Internet in Medicine. 2006 Mar;31(1):45-52. doi: 10.1080/14639230600552601.
253. Beck F, Richard J-B, Nguyen-Thanh V, Montagni I, Parizot I, Renahy E. Use of the Internet as a health information resource among French young adults: Results from a nationally representative survey. Journal of Medical Internet Research. 2014;16(5):193-205. doi: 10.2196/jmir.2934. PMID: 24824164.
254. Butrick M, Roter D, Kaphingst K, Erby LH, Haywood C, Jr., Beach MC, et al. Patient reactions to personalized medicine vignettes: an experimental design. Genetics In Medicine: Official Journal Of The American College Of Medical Genetics. 2011;13(5):421-8. PMID: 21270639. doi: 10.1097/GIM.0b013e3182056133.
255. Cox A, Faithfull S. Aiding a reassertion of self: a qualitative study of the views and experiences of women with ovarian cancer receiving long-term nurse-led telephone follow-up. Supportive Care in Cancer. 2015;23(8):2357-64. PMID: 25588575. doi: 10.1007/s00520-014-2578-4.
256. Engler J, Adami S, Adam Y, Keller B, Repke T, Fügemann H, et al. Using others' experiences. Cancer patients' expectations and navigation of a website providing narratives on prostate, breast and colorectal cancer. Patient Education and Counseling. 2016;99(8):1325-32. PMID: 27067064. doi: 10.1016/j.pec.2016.03.015.
257. Geuens J, Swinnen TW, Westhovens R, de Vlam K, Geurts L, Vanden Abeele V. A Review of Persuasive Principles in Mobile Apps for Chronic Arthritis Patients: Opportunities for Improvement. Jmir Mhealth and Uhealth. 2016 Oct-Dec;4(4):15. PMID: 27742604. doi: 10.2196/mhealth.6286.
258. Herian MN, Shank NC, Abdel-Monem TL. Trust in government and support for governmental regulation: the case of electronic health records. Health Expectations: An International Journal Of Public Participation In Health Care And Health Policy. 2014;17(6):784-94. PMID: 22809222. doi: 10.1111/j.1369-7625.2012.00803.x.
259. Katz JE, Roberge D, Coulombe G. The cancer patient's use and appreciation of the internet and other modern means of communication. Technology In Cancer Research & Treatment. 2014;13(5):477-84. PMID: 24000994. doi: 10.7785/tcrtexpress.2013.600267.
260. Liu CF, Tsai YC, Jang FL. Patients' Acceptance towards a Web-Based Personal Health Record System: An Empirical Study in Taiwan. International Journal of Environmental Research and Public Health. 2013 Oct;10(10):5191-208. PMID: 24142185. doi: 10.3390/ijerph10105191.
261. Mills R, Barry W, Haga S. Public Trust in Genomic Risk Assessment for Type 2 Diabetes Mellitus. Journal of Genetic Counseling. 2014;23(3):401-8. PMID: 24292896. doi: 10.1007/s10897-013-9674-3.
262. Mou J, Shin DH, Cohen JF. Tracing College Students' Acceptance of Online Health Services. Int J Hum-Comput Interact. 2017;33(5):371-84. doi: 10.1080/10447318.2016.1244941.
263. Spil T, Klein R. The personal health future. Health Policy and Technology. 2015;4(2):131-6. doi: 10.1016/j.hlpt.2015.02.004.
264. Teixeira PA, Gordon P, Camhi E, Bakken S. HIV patients' willingness to share personal health information electronically. Patient Education And Counseling. 2011;84(2):e9-e12. PMID: 20724095. doi: 10.1016/j.pec.2010.07.013.

265. Crawford J, Larsen-Cooper E, Jezman Z, Cunningham SC, Bancroft E. SMS versus voice messaging to deliver MNCH communication in rural Malawi: assessment of delivery success and user experience. Global Health, Science And Practice. 2014;2(1):35-46. PMID: 25276561. doi: 10.9745/GHSP-D-13-00155.
266. Deng ZH, Mo XT, Liu S. Comparison of the middle-aged and older users' adoption of mobile health services in China. International Journal of Medical Informatics. 2014 Mar;83(3):210-24. PMID: 24388129. doi: 10.1016/j.ijmedinf.2013.12.002.
267. Hansen EH, Hunskaar S. Understanding of and adherence to advice after telephone counselling by nurse: a survey among callers to a primary emergency out-of-hours service in Norway. Scandinavian journal of trauma, resuscitation and emergency medicine. 2011;19:48. PMID: 21892945.
268. Kahane S, Stutz E, Aliarzadeh B. Must we appear to be all-knowing? Patients' and family physicians' perspectives on information seeking during consultations. Canadian Family Physician. 2011;57(6):e228-e36. PMID: 21673199.
269. Levy S, Bradley DA, Morison MJ, Swanston MT, Harvey S. Future patient care: tele-empowerment. Journal Of Telemedicine And Telecare. 2002;8 Suppl 2:52-4. PMID: 12217135.
270. Lorence DP, Park H. Measuring dissimilarity in online health search activities. Technology And Health Care: Official Journal Of The European Society For Engineering And Medicine. 2006;14(2):79-89. PMID: 16720951.
271. Serrano KJ, Yu M, Riley WT, Patel V, Hughes P, Marchesini K, et al. Willingness to Exchange Health Information via Mobile Devices: Findings From a Population-Based Survey. Annals of Family Medicine. 2016 Jan-Feb;14(1):34-40. PMID: 26755781. doi: 10.1370/afm.1888.
272. Chung JE. Social interaction in online support groups: Preference for online social interaction over offline social interaction. Computers in Human Behavior. 2013 Jul;29(4):1408-14. doi: 10.1016/j.chb.2013.01.019.
273. Ek S, Eriksson-Backa K, Niemelä R. Use of and trust in health information on the Internet: a nationwide eight-year follow-up survey. Informatics For Health & Social Care. 2013;38(3):236-45. PMID: 23514041. doi: 10.3109/17538157.2013.764305.
274. Driessen J, Bonhomme A, Chang W, Nace DA, Kavalieratos D, Perera S, et al. Nursing Home Provider Perceptions of Telemedicine for Reducing Potentially Avoidable Hospitalizations. Journal Of The American Medical Directors Association. 2016;17(6):519-24. PMID: 26969534. doi: 10.1016/j.jamda.2016.02.004.
275. Demiris G, Speedie S, Finkelstein S. A questionnaire for the assessment of patients' impressions of the risks and benefits of home telecare. Journal Of Telemedicine And Telecare. 2000;6(5):278-84. PMID: 11070589.
276. Cates JR, Ortiz RR, North S, Martin A, Smith R, Coyne-Beasley T. Partnering with middle school students to design text messages about HPV vaccination. Health Promotion Practice. 2015;16(2):244-55. PMID: 25258431. doi: 10.1177/1524839914551365.
277. Chen X, Zhang X. How Environmental Uncertainty Moderates the Effect of Relative Advantage and Perceived Credibility on the Adoption of Mobile Health Services by Chinese Organizations in the Big Data Era. International Journal Of Telemedicine And Applications. 2016;2016:3618402-. PMID: 28115932. doi: 10.1155/2016/3618402.
278. French RS, McCarthy O, Baraitser P, Wellings K, Bailey JV, Free C. Young People's Views and Experiences of a Mobile Phone Texting Intervention to Promote Safer Sex

Behavior. JMIR Mhealth And Uhealth. 2016;4(2):e26-e. PMID: 27083784. doi: 10.2196/mhealth.4302.
