# Supplementary material for: Elements of Trust in Digital Health Systems: Scoping Review
Source: J Med Internet Res. 2018 Dec 13;20(12):e11254. doi: 10.2196/11254 (PMC6315261; doi:10.2196/11254)
Supplement: Multimedia Appendix 3 [file jmir_v20i12e11254_app3.pdf]

Appendix 3: List of Study Countries

Country	Total Number of Articles
Australia	16
Austria	1
Bangladesh	1
Canada	15
China	8
Czech Republic	1
Denmark	2
European Union	1
Finland	2
France	1
Gambia	1
Germany	4
Greece	1
Guatemala	1
Ireland	3
Israel	1
Italy	1
Iran	1
Japan	2
Latvia	1
Malawi	1
Mexico	1
Mozambique	1
Nepal	1
Netherlands	6
New Zealand	2
Norway	10
Poland	1
Portugal	1
Romania	1
South Africa	5
South Korea	1
Spain	4
Sri Lanka	1
Sweden	12
Switzerland	1
Taiwan	8
Tanzania	1
Uganda	1
United Kingdom	47
United States	101
