# Supplementary material for: Elements of Trust in Digital Health Systems: Scoping Review
Source: J Med Internet Res. 2018 Dec 13;20(12):e11254. doi: 10.2196/11254 (PMC6315261; doi:10.2196/11254)
Supplement: Multimedia Appendix 4 [file jmir_v20i12e11254_app4.pdf]

Appendix 4: Health Technology Types

Digital Health category	Technologies and Services
**EHRs**	Computer-Based Patient Record (CBPR), Computerized Decision Support Systems (CDSS), Computerized Physician Order Entry (CPOE), Computerized Provider Documentation (CPD), e-prescribing, Electronic Health Care Records (EHCR), Electronic Medical Records (EMR), Electronic Patient Records (EPR), Health Information Exchange (HIE), Patient Access Electronic Record System (PAERS), Personal Health Records (PHR), Google Health, Microsoft Vault, Personal Medical Summaries, Personally Controlled Electronic Health Records (PCEHR), Personally Controlled Health Records (PCHR), Physician Order Entry (POE), Picture Archiving Communications Systems (PACS), Primary Health Information System (PHIS), Summary Care Records (SCR), Virtual Lifetime Electronic Records (VLER)
**Health IT**	Automated Medication Dispensing System (AMDS), Digital interactive TV (Di-TV), e-prescribing systems, health websites, online consultations, online health information, online interventions, Online Support Groups (OSG), Personal Digital Assistant (PDA), web-based interventions, web-based medical Service, Electronic Clinical Support System
**Telehealth/Telemedicine**	telephone-based follow-up, distant learning, internet Mediated Cognitive Behavioral Therapy, online counselling & therapy, remote monitoring, teledermatology, telemedicine portals, telemonitoring system, telenursing, telephone consultations, telepsychotherapy,

	telerehabilitation, videoconferencing, virtual clinics
**Wearable Devices**	Activity trackers, smart glasses (google glass)
**Personalized Medicine**	biobanks, commercial websites, data sharing, Direct to Consumer Genetic-Testing (DTC G-T), genetic blogs, Genetically Personalized Medication (GMP), Genome-Wide Association Studies (GWAS), Genomic research, pharmacogenomics, Whole Genome Sequencing (WGS)
**mHealth**	mobile computing devices, mobile health services, mobile job aid, Short Message Service (SMS), smartphone applications, text messaging, voice messages
